# Synthetic biology for sustainable abundance: biomining, electromicrobial production, and hyper-engineerable microbes

**DOI:** 10.1039/d6cc01792d

**Published:** 2026-10-22

**Authors:** Gretchen Vengerova, Buz Barstow

**Affiliations:** a Department of Biological and Environmental Engineering, Cornell University Buz Barstow, 228 Riley-Robb Hall Ithaca NY 14853 USA bmb35@cornell.edu

## Abstract

Over the next few decades, there is a compelling need to build a new sustainable energy infrastructure to replace the fossil fuel system that we have relied on since the 1700s. This infrastructure must not only replace what exists but also far exceed its capabilities, delivering more power to more people to meet the growing demands from both developing and advanced economies. But how do we increase energy use without harming the Earth? Biology drives the Earth's geochemical cycles, operating with far greater elemental and energy fluxes than all of human civilization. This suggests that synthetic biology could solve at least some of our environmental problems while providing access to much greater energy. However, leveraging biology remains a formidable challenge, in part because we do not yet understand nearly enough about the basic science underlying the biological systems that bring the most unusual and useful capabilities to sustainability and energy. This challenge is not unique to biology and sustainability – groundbreaking technologies of the past have required not only curiosity-driven basic research but also use-inspired basic research. Over the past decade, our lab has discovered new basic science needed to build synthetic biology technologies and established itself as a leader in sustainability and synthetic biology. This review summarizes breakthroughs from our lab over the past decade in three key areas: biomining, electrically driven metabolism, and hyper-engineerable microbes.

## Abstract 2

Engineered microbes could mine the metals the energy transition needs, lock up CO_2_ as stone, and turn renewable electricity into fuels and food. This review covers almost a decade of fundamental biology needed to build them.

## Graphical abstract

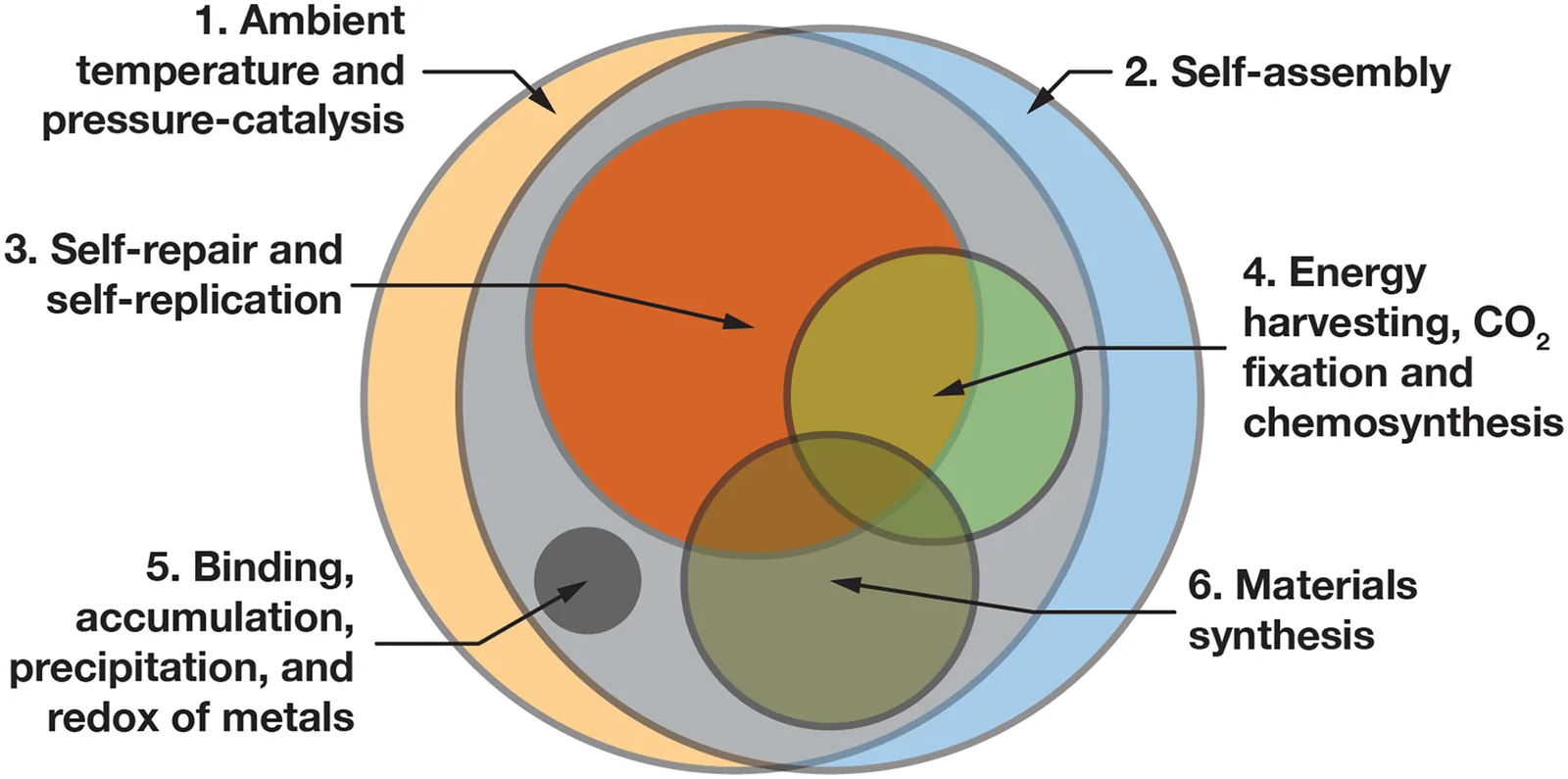

## 1. Introduction

If we look back through history, or around the world today, we can see there is a clear connection between energy use per capita, per capita income,^1^ and quality of life as measured by life expectancy or human development.^2–5^ At the top end of the scale, there is a legitimate argument about which country has the best quality of life and allows the most human flourishing: is it the United States, whose citizens have amongst the highest per capita energy use and income in the world alongside an incredibly entrepreneurial and dynamic population, but has a life expectancy notably lower than other advanced nations, even for its most privileged members?^6^ Is it Japan, whose citizens use 50% of the energy that an average American does,^1^ but whose life expectancies are amongst the longest on Earth^2^ and are the inheritors of rich, globally admired culture? Or is it the UK, whose citizens use only 37% of the energy used by the average American, is a leader in green energy deployment, boasts an open multi-cultural society, and is a dynamic creator of modern culture? The answers to these questions are not clear and certainly will not be answered in this article. But ask yourself: would you rather live in one of these advanced nations (energy use per capita in the G7 nations differs by no more than a factor of 3.5, between the UK and Canada), or live in the low-income countries (*e.g.*, Afghanistan, Somalia, and South Sudan) whose citizens use on average only 2% of that used in high-income nations^1^ (Dataset S1)? A clear connection between life satisfaction and GDP per capita exists, suggesting that the answer is the former.^7^

Increased energy use does not automatically improve social conditions and quality of life; it only creates the potential for it. For example, consider a scenario where the United States re-shores heavy industries and manufacturing, dramatically increasing energy consumption while using current technologies. This approach would increase CO_2_ emissions and cause air, water, and soil pollution. Additionally, without appropriate distribution mechanisms, the prosperity and benefits created could be concentrated in the hands of only a few. But, imagine another future version of the world where energy use per capita is also twice what it is today. This energy could be used to build, heat, and cool much bigger living spaces to ease some of the pressures of family life, operate ultra-high speed trains and flying cars, build and operate advanced robotic factories, enable the use of advanced building materials to preserve local architectural beauty while allowing a mass expansion of housing stock, and water desalination to end water shortages.

Over the past few years, concerns about stagnation of technological and economic progress^8,9^ have started to challenge concerns about environmental degradation and climate change that have dominated public discourse since the early 1970s.^10,11^ This raises the question: how does a country or an entire civilization increase its use of energy, and all of the good things that come with it, without despoiling its environment?

The physicist John Barrow noted that as our civilization advances, the precision with which we can control matter grows alongside energy use.^12^ For example, in the late 1700s, the frontier of engineering was boring cylinders for cannon and steam engines with a precision that “does not err the thickness of an old shilling at any part” (0.05 to 0.1 inches, or 1.2 to 2.5 millimeters).^13^ Today, 250 years later, feature sizes of only a few nanometers can be achieved with extreme ultraviolet lithography.^14^ Barrow suggested that more advanced civilizations might exert even more precise control over matter.^12^

To increase energy use without harming the environment, we think that we will have to take increasingly precise control over matter, and that synthetic biology is the right tool for this. It can be said that biology combines atomic precision with planetary scale. Nature gives a great existence proof for this: the biosphere today channels ≈7× as much power as all of human civilization (see Note S1), with no human intervention and at no cost, using catalysts that self-replicate and self-assemble with sub-angstrom precision to perform highly selective catalysis under ambient conditions.^15^ Today, protein structure prediction tools allow the positions of atoms within *de novo* protein molecules to be predicted to within ≈1 Å.^16^ A diagram showing some of the capabilities of biology and their applications is shown in Fig. 1. We think that in the future, technologies will either draw inspiration from these features of biology or directly harness biology through advanced genetic engineering. We are not the only people to think this: advanced biotechnologies have been the subject of both science fiction^17–22^ and serious scientific speculation^23,24^ for decades. Synthetic biology's ambitions extend well beyond sustainability. The field also envisions engineering biology for the synthesis of materials with novel and extreme properties,^25–29^ cell-free manufacturing,^30^ regenerative medicine,^31^ and advanced medicines.^32–34^ However, we still believe, as we argued in 2017,^15^ that applying biology to health and to high-value molecules, and applying it at the ultra-large scales needed to solve our sustainability problems, are not just different in degree, but different in kind. That is why, in the same article, we laid out a roadmap for using biology to solve problems in climate and energy.^15^ While we admit it is unlikely that every new energy technology will be biological in nature within our lifetimes, we would be even more surprised if at least one new major energy technology does not use engineered biology in some form or another by the end of the century (as an aside, biomass is already the world's largest source of renewable energy^35^). But, harnessing the potential of biology remains an enormous challenge: we do not understand nearly enough about the genetics and mechanisms of unusual biology that offers the most promising capabilities for applications in energy and sustainability.^36^ Transformational technologies typically require not only curiosity-driven basic research but also use-inspired basic research that simultaneously provides fundamental insights into nature and drives application.^37^

**Fig. 1:**
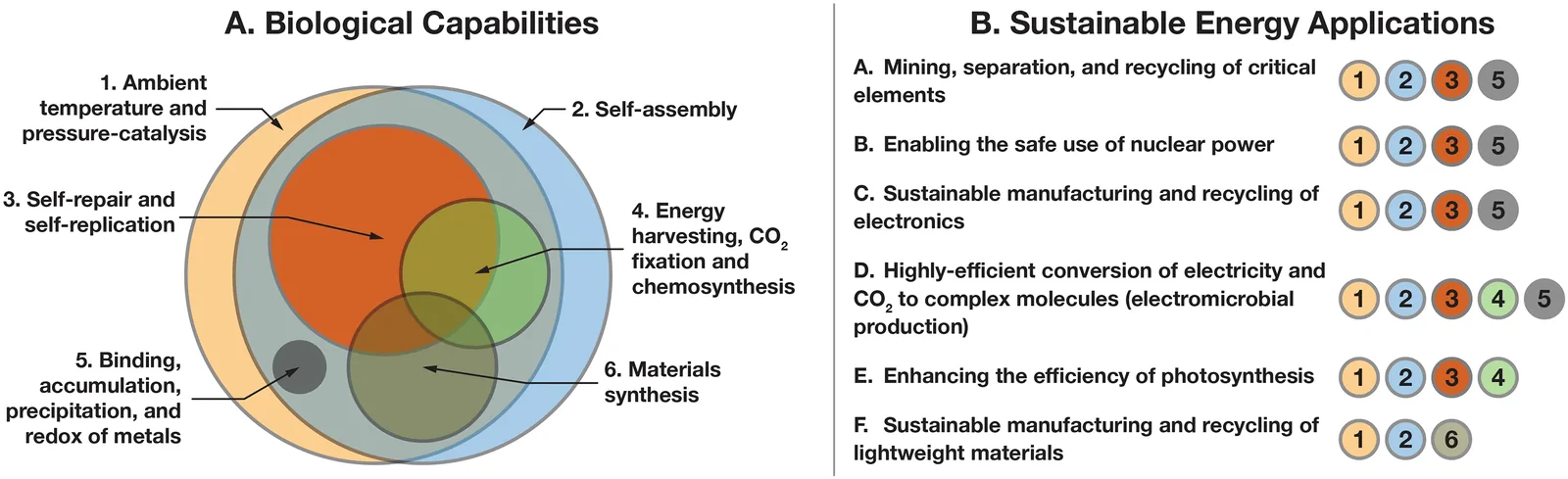
(A) Biological capabilities relevant to sustainable energy and (B) sustainable energy applications. Alongside each potential application we have noted the most useful biological capability for that application followed by additional capabilities. Adapted from Adesina *et al.*^15^ with permission from Elsevier, copyright 2017.

This article summarizes the progress our lab has made in three areas over the past eight years: (1) biomining for the metals we will need for sustainable energy technologies like wind turbines, electric vehicles, electro-catalysts, high-temperature superconductors, and CO_2_ mineralization; (2) electromicrobial production for the conversion of CO_2_ and renewable electricity into complex molecules like food, fuel, and commodity chemicals; and (3) the development of highly engineerable microbes.

## 2. Biomining and CO_2_ sequestration

### 2.1. Introduction to biomining

The area of our research program that has seen the fastest development is biomining. Our lab is genetically engineering microbes for the environmentally friendly mining of metals for sustainable energy technologies and ultra-large scale CO_2_ sequestration.

The revolution triggered by the transition from fossil fuels to renewable energy will create an unprecedented demand for energy-critical minerals and elements. Metals are critical ingredients for present and future sustainable energy technologies including solar,^38,39^ catalysts,^38,39^ wind turbines,^39,40^ electric vehicles,^38,40^ batteries,^38,41^ superconductors,^42^ nuclear reactors, high-strength lightweight alloys,^43^ and high efficiency electronics. Use of nickel, rare earth elements (REEs) and cobalt will increase by more than 50% to 70% by 2040 (ref. 44) (Fig. 2A). Total demand for nickel, cobalt, copper, platinum group elements (PGEs), gold, silver, and zinc is expected to exceed 3 billion tonnes in response to the clean energy transition.^38^ On top of this, metal demand will be further increased by advanced defense technologies like drones, precision weapons and sensors,^45–47^ and by advanced electronics for artificial intelligence.^44,48^ Over the next few decades, the increase in demand for metals will exceed the total amount mined over all of previous human history.^49^ This will require non-traditional metal sources,^49^ including previously untapped low-grade ores and extensive recycling.^50^ Traditional extraction and separation technologies, especially when applied to non-traditional sources, pose extreme environmental challenges.^51–53^ On top of this, as the supply chains for many metals stretch across the globe, there is significant risk of disruption by bad actors.

**Fig. 2:**
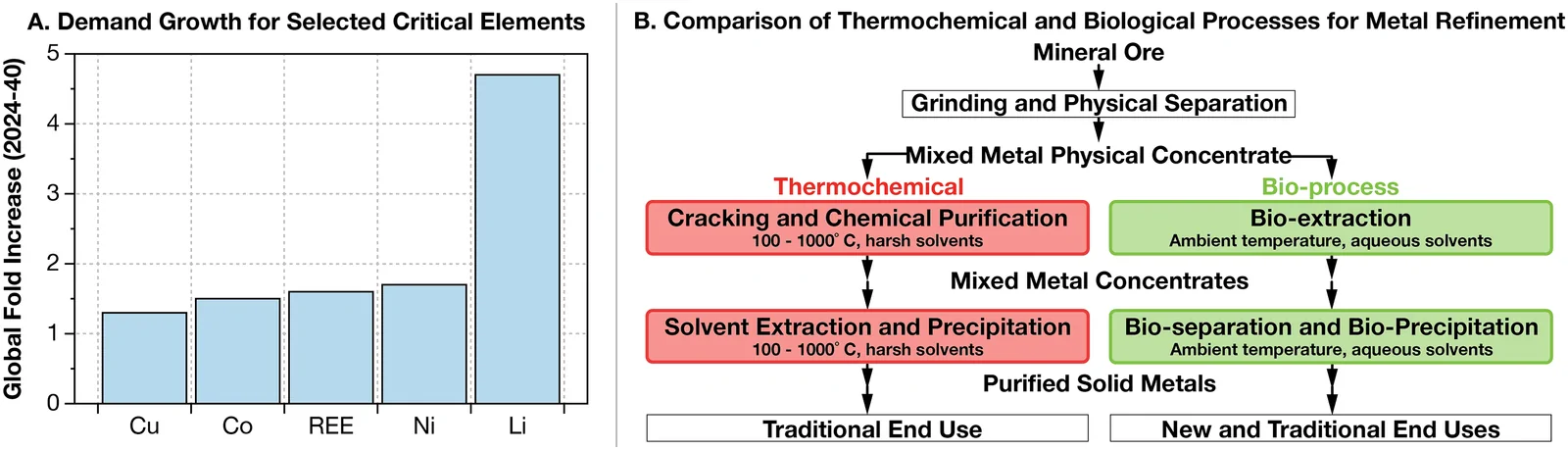
Building a new sustainable energy infrastructure will require a dramatic increase in metal supply that will come with major environmental impacts if delivered by traditional mining. (A) Projected increase in global demand for copper, cobalt, rare earth elements, nickel, and lithium from 2024 to 2040 under the International Energy Agency's Stated Policies Scenario (STEPS).^44^ (B) Biological processes could replace many of the most harmful steps of mining. Furthermore, bioprocesses like nanoparticle precipitation could even provide completely new advanced products.

Biomining has the potential to increase the efficiency and sustainability of mining processes (Fig. 2B). It has already revolutionized the supply of copper and gold from low-grade sulfidic ores.^54^ Today, 12% (ref. 55) to 20% (ref. 54) of the world's copper and about 5% of its gold come from a redox-mediated biological process performed by the acidophilic microbe *Acidithiobacillus ferrooxidans*.^54^ This process is uniquely well-suited for extracting metals from low grade ores. This means that more metal can be extracted from the same amount of rock, reducing the need for excavation, and reducing the environmental impact of mining. This increases the chances that these metals can be mined and refined within the environmental standards of the advanced nations, reducing the possibility that the negative externalities of sustainable energy technologies will be felt by the less technologically advanced nations, and increasing the resilience of the supply chains for these metals.

But, right now there are no industrially useful microbes for biomining from recycled feedstocks (*e.g.*, electronic, magnet, and battery waste) or from other ore-types (*e.g.*, carbonates, phosphates, and silicates). To get microbes to mine all of the other metals that will be needed for the sustainability revolution, we will need to build them with advanced genetic engineering.

Biology has at least six (sometimes overlapping) methods of interacting with metals and minerals that can be harnessed for biomining: acid production;^56^ redox change;^57^ chelation;^58–60^ hyper-accumulation;^61^ biosorption;^62^ and precipitation (while often a product of redox change, it can occur without redox change, for example by reaction with CO_2_)^63^ (Fig. 3). Out of these, acid production, redox change, and hyper-accumulation can be used for metal extraction. Redox change, chelation, hyper-accumulation, biosorption, and precipitation can be used for metal separation. However, our understanding of the genetics underlying these processes remains insufficient to harness them for biomining.

**Fig. 3:**
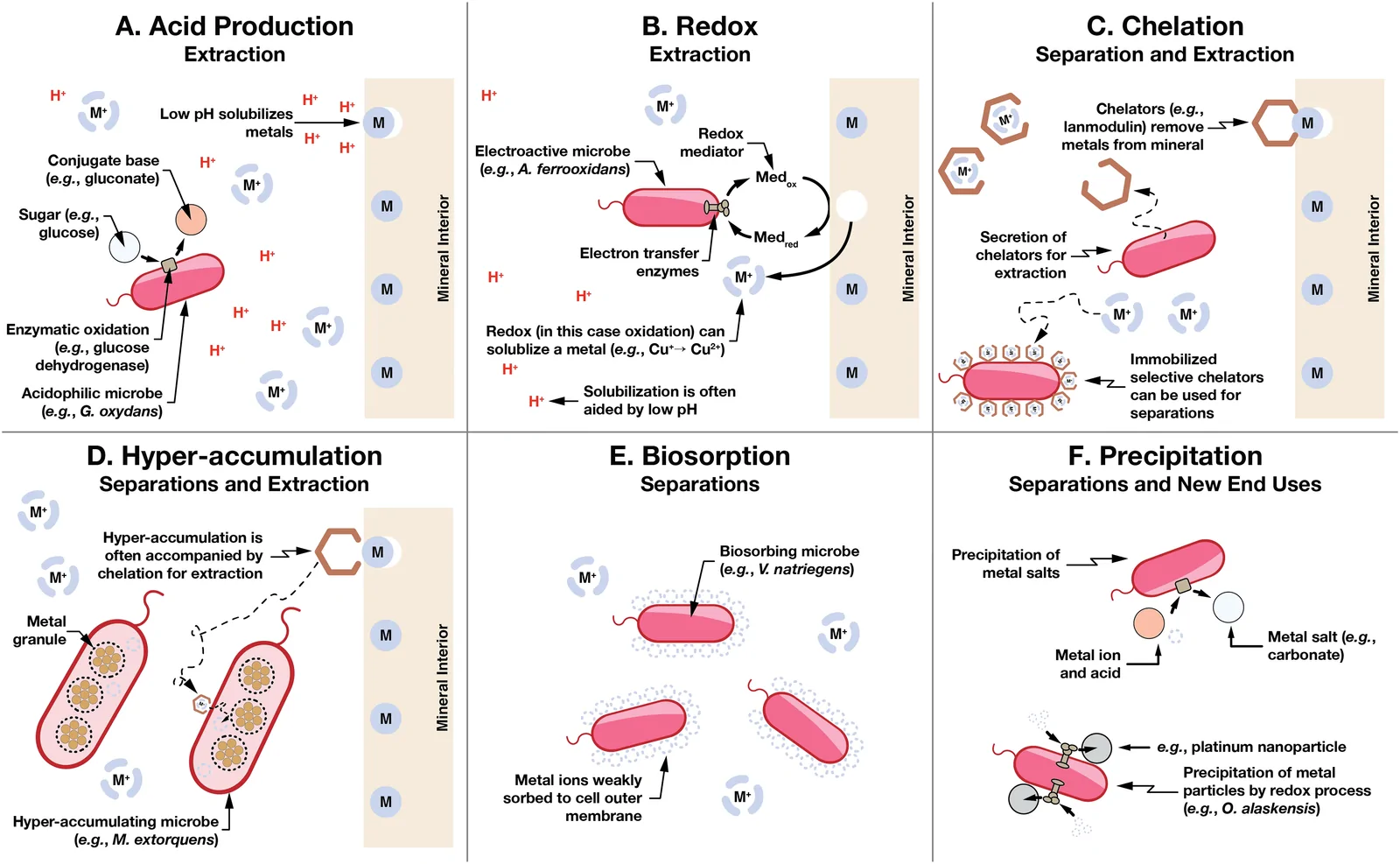
Nature gives at least six different microbial mechanisms for interaction with metals and minerals that can be used for metal extraction and separation and new end uses of metals. (A) Microbes like *Gluconobacter oxydans* are capable of oxidizing sugars to bio-degradable organic acids to solubilize metals.^78,80^ In addition, microbes like *Acidithiobacillus ferrooxidans* can oxidize sulfides to produce sulfuric acid.^186^ (B) Electroactive microbes can reduce or oxidize metals to solubilize them, either through the use of redox shuttles or directly. Acidophilic microbes like *A. ferrooxidans* can use iron ions at low pH (both Fe^2+^ and Fe^3+^ are soluble at low pH) as redox shuttles to oxidize Cu and solubilize it from chalcopyrite.^186^ Similarly, neutrophilic microbes like *Shewanella oneidensis* can reduce Fe^3+^ to Fe^2+^ to solubilize it at neutral pH.^187–189^ (C) Microbes like *Methylorubrum extorquens* can release highly selective metal chelators that can remove metals (in this case lanthanides) from minerals.^61,190,191^ Meanwhile, many microbes use siderophores to extract iron from silicate minerals and dissolve them.^192,193^ (D) Often chelators are used to transport metals, in this case lanthanides, to microbes, including *Methylorubrum extorquens* and related species, where they are hyper-accumulated inside intracellular granules.^61,190,191^ (E) Many biological materials including microbial biomass^108^ are capable of weakly sorbing metals onto their surfaces. Some microorganisms including *Roseobacter* sp. AzwK-3b, *S. oneidensis* and *V. natriegens* can even selectively biosorb lanthanides,^62,110,112^ and this selectivity can even be improved by genetic engineering.^112^ (F) Microbes such as *Oleidesulfovibrio alaskensis* can precipitate metals such as platinum and palladium to produce nanoparticles. These nanoparticles sometimes have superior properties to chemically produced nanoparticles.^194^ Inspired by a drawing by Michael Capeness and Louise Horsfall.

### 2.2. Genetically engineered microbes for bioleaching rare earth elements

Rare earth elements (widely accepted to include scandium, yttrium and the lanthanide series) (REEs) are vital ingredients of sustainable energy, advanced defense, and electronic technologies (Table S1). Scandium is essential for lightweight high-strength alloys,^43,64^ yttrium is a potential ingredient of future high-temperature superconductors,^65^ while the lanthanides are used in high-strength lightweight magnets for electric vehicles and wind turbines,^40,66,67^ nuclear reactor moderators,^68,69^ and novel electromagnetic amplification applications.^70,71^

However, despite their vital role in sustainability technologies, current industrial methods for extracting REEs from ore and subsequently refining them into individual elements are deeply environmentally damaging^72,73^ (Fig. 2B). As a result of the high environmental standards of advanced nations, the world's supply of refined REEs comes almost exclusively from China.^74,75^ This has inspired both our lab and other research groups to develop new biotechnologies for biomining REEs.^76,77^

A promising solution to the environmental impact of REE-extraction is bioleaching.^72,78–82^ In this process, microbes secrete a mineral-dissolving cocktail called a biolixiviant which contains organic acids and other biologically synthesized molecules. However, the efficiency of REE-bioleaching is often low, making it uncompetitive with thermochemical processes (≈3–5%^83^ for *Aspergillus* species on monazite ore *vs.* ≥89–98%^84,85^). The bacterial species *Gluconobacter oxydans* is particularly efficient at recovering REEs from spent fluid cracking catalysts (FCCs) (≈50%),^78^ one of the lowest cost REE feedstocks. A techno-economic analysis of a *G. oxydans* bioleaching process to extract REEs from spent FCCs demonstrated a small margin of profit, but improvement of the bioleaching mechanisms was necessary for broad commercial application.^86^

Almost seven years ago, we hypothesized that we could engineer *G. oxydans* to make it dissolve rocks much more efficiently. Even though we could edit *G. oxydans*’ genome, no one knew where to do it. This problem is widespread in synthetic biology and will be discussed in depth later in this article. Using Knockout Sudoku^87,88^ (Fig. 4), we developed the first whole genome knockout collection (a collection of clonally isolated single-gene knockout mutants, one for each non-essential gene in the genome) of *G. oxydans* and performed the first characterization of the genes involved in biolixiviant production.^80^ In total we were able to find 165 genes that controlled acid production using high throughput screens.^80^

**Fig. 4:**
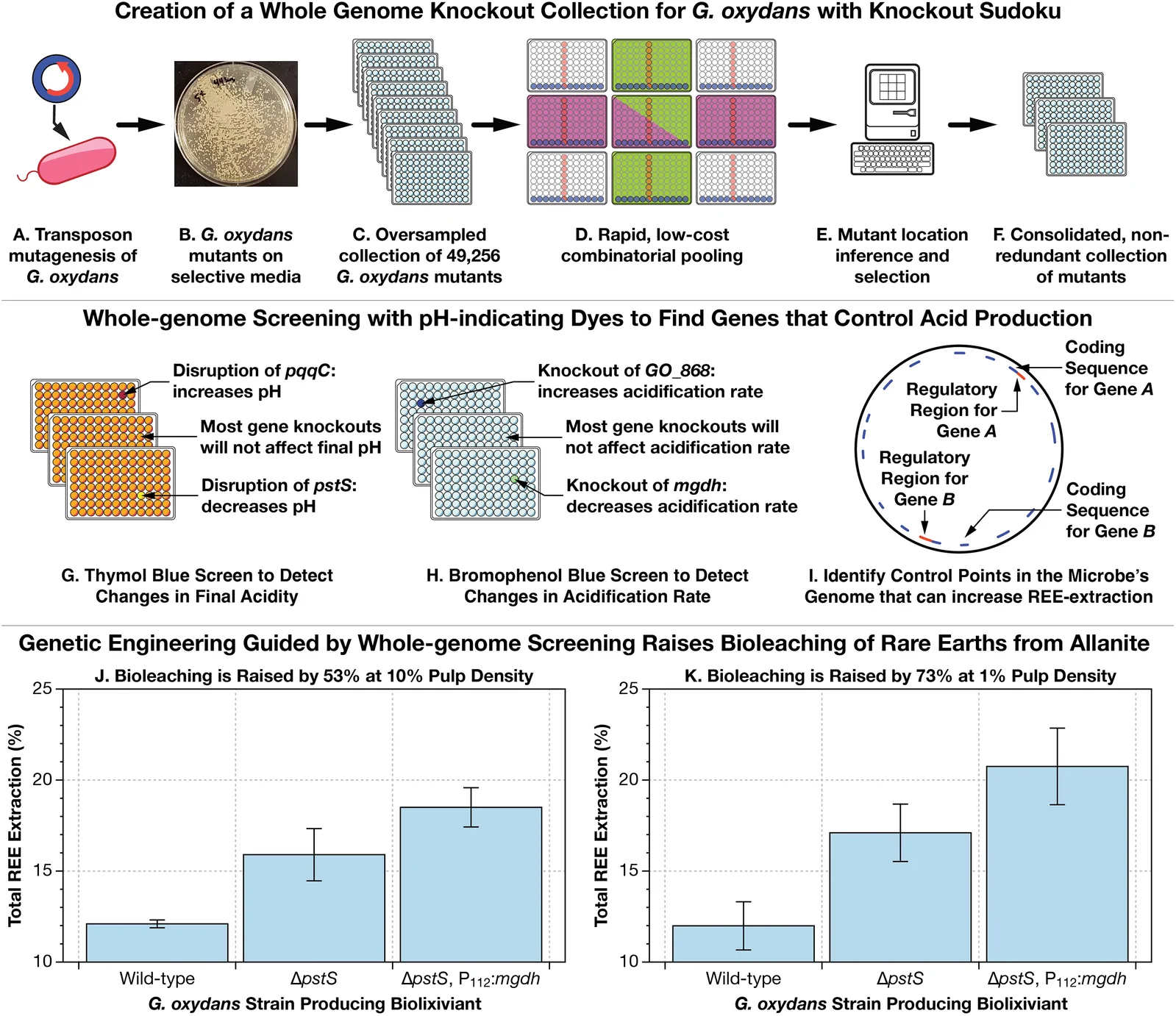
Whole genome screening allowed us to engineer the mineral-dissolving microbe *Gluconobacter oxydans* to increase acid production and dissolve rare earth element containing minerals. (A–F) First, we used the combinatorial pooling system Knockout Sudoku^87,88^ to rapidly build a whole genome knockout collection for *G. oxydans* at low cost.^80^ (A–C) An extremely large collection of random gene disruption mutants are created by transposon mutagenesis. (D) All mutants are combined into a single next-generation sequencing library that is barcoded to allow the location of the mutants to be identified by a Bayesian inference algorithm (E). (E and F) Finally, a set of mutants containing a representative for every non-essential gene in the genome is selected and built. (G and H) A high-throughput screen was used to search for genes that control acid production by *G. oxydans*. (I) The regulatory sequences of genes that control acid production were edited to increase acid production. Genes like *mgdh* whose knockout reduced acid production were up-regulated, while genes whose knockout increased acid production like *pstS* were deleted or down-regulated. (J and K) Extraction of REE from the mineral allanite was increased by 53% by a mutant of *G. oxydans* with deleted *pstS* and the *mgdh* gene up-regulated with the P_112_ promoter at 10% pulp density, and 73% at 1% pulp density.^81^

We identified two systems in *G. oxydans* that have opposing effects on REE-bioleaching. Knocking out the phosphate signaling and transport (*pst*) system takes the brakes off production. Disruption of the *pstC* gene raises REE-extraction from retorted phosphor powder by 18%. On the other hand, disrupting the *mgdh* gene that encodes the membrane-bound glucose dehydrogenase that converts glucose to gluconic acid, or genes involved in the pathway for synthesis of its pyrroloquinoline quinone (PQQ) cofactor (the *pqq* and *tld* operons), can reduce bioleaching by over 99% (Table 1).^80^

**Table 1:** Microorganisms, engineering approaches and applications discussed in this review

Organism	Engineering approach	Application	Key result	Ref.
*Acidithiobacillus ferrooxidans*	None (naturally occurring)	Bioleaching of copper and gold from low-grade sulfidic ores	12–20% of world copper supply, ≈5% of gold	54 and 55
*Aspergillus* spp.	None (naturally occurring)	REE bioleaching from monazite ore	3–5% extraction, against 89–98% for thermochemical routes	83–85
*Gluconobacter oxydans*	None (naturally occurring)	REE bioleaching from spent fluid cracking catalyst	≈50% extraction; techno-economic analysis shows narrow margin	78 and 86
*G. oxydans*	Whole-genome knockout collection by Knockout Sudoku; high-throughput acid-production screen	Identification of genes controlling biolixiviant production	165 genes controlling acid production identified	80
*G. oxydans*	Disruption of *pstC* (phosphate signaling and transport)	REE extraction from retorted phosphor powder	18% increase in extraction	80
*G. oxydans*	Disruption of *mgdh*, and of *pqq* and *tld* operons	Mechanistic dissection of acid-mediated bioleaching	>99% reduction in bioleaching, confirming the role of gluconic acid	80
*G. oxydans*	Systems-biology-guided multi-gene engineering	REE bioleaching from neodymium phosphate	≈1200% increase in bioleaching	81 and 82
*G. oxydans*	Reduced-redundancy knockout collection (85% smaller, 100% complete); Arsenazo-III competitive assay	Discovery of non-acid bioleaching mechanisms	68 genes affecting bioleaching with negligible pH change; *GO_1096* knockout reduces bioleaching by 95% at ΔpH 0.2; up-regulation raises bioleaching by 64%	82
*Shewanella oneidensis* MR-1	Whole-genome knockout collection screen	First comprehensive genetic profile of REE biosorption	242 genes controlling biosorption; 9 genes alter selectivity by 1–4%, modeled to shorten enrichment by up to 27%	110
*S. oneidensis* MR-1	Modeled multi-locus edits (3–4 of 9 genes)	REE separation process design	Predicted ≈90% reduction in separation process length	111
*Vibrio natriegens*	Multiple rounds of *in vivo* random mutagenesis and selection	REE biosorption and separation	210% increase in biosorption capacity; up to 50% selectivity gain between La and Lu; 15% selectivity gain between adjacent heavy REEs (Tm/Yb), leapfrogging small-molecule separation	112
*G. oxydans*	None (naturally occurring); cellulosic-biomass-derived sugar feedstock	Ultramafic bioleaching for CO_2_ mineralization and Ni/Co recovery	Best performer of the panel tested; biolixiviant effectiveness rises with pulp density	122 and 123
*Sphingomonas desiccabilis*, *Penicillium simplicissimum*	None (naturally occurring)	Ultramafic bioleaching	Lower weathering performance than *G. oxydans*	123
*S. oneidensis* MR-1	Whole-genome knockout collection by Knockout Sudoku	Genome-scale genetic platform for a non-model electroactive microbe	≈99% of non-essential genes covered for ≈$10 000, against ≈$4 M for comparable collections	87 and 88
*S. oneidensis* MR-1	AHDS_red_ oxidation screen of knockout collection; bio-electrochemical validation	Discovery of key genes in an extracellular electron uptake pathway	149 genes associated with EEU; 5 indispensable for cathodic uptake, 4 of which do not affect anodic donation	164
*Cupriavidus necator*	None (naturally occurring H_2_-oxidizing autotroph)	H_2_-mediated electromicrobial production	Solar-to-chemical efficiency exceeding the theoretical maximum of photosynthesis	140
*C. necator*	Coupling to CdSe quantum dots; single-cell and single-molecule imaging	Direct extracellular electron uptake by H_2_-oxidizing microbes	≈1 nA uptake, ≈1000× higher than predicted; hydrogenases shown to accept electrons directly	174
*Escherichia coli*	Engineered CO_2_ fixation; formate assimilation; heterologous Mtr complex	Reference chassis for combining extracellular electron uptake with carbon fixation	Each capability demonstrated separately; combination not yet achieved	175–178
*V. natriegens*	Genomic integration of *tfoX* from *Vibrio cholerae*; minimal competence media	Hyper-engineerable chassis for EMP and biomining	Natural competence sustained for tens of hours, enabling synthetic biology with no capital equipment	181

These new genetic discoveries on bioleaching gave us a roadmap for making a better REE-biomining microbe. Using them, we have enhanced the REE-bioleaching capability of *G. oxydans* by almost 1200% from neodymium phosphate.^81,82^

However, acid production is only part of the story of REE-bioleaching. Almost a decade ago, David Reed and coworkers at Idaho National Laboratory found that *G. oxydans* was more effective at extracting REEs from spent FCC than gluconic acid alone, which is the primary component of its biolixiviant.^78,89^ This suggested to us that *G. oxydans* employs additional metal extraction mechanisms (*e.g.*, chelation, hyper-accumulation, or redox change) that amplify the effectiveness of the acid. If these mechanisms were discovered, they could be harnessed to make bioleaching much more efficient. However, it would be almost impossible to find the genes that encoded these mechanisms with the acid production assays that we had developed for our first screens of our *G. oxydans* knockout collection.

While acid production is a useful proxy for metal extraction, it is insufficient to measure extraction by all methods. To solve this problem, we adapted a competitive assay with the lanthanide-chelating dye Arsenazo-III^90^ to measure extraction from artificially synthesized neodymium phosphate (colloquially referred to as synthetic monazite)^81^ (Fig. 5A). Performing this assay is a great example of why connecting genotype to non-fitness-related phenotypes is such a challenge: it is time-consuming and expensive. To make this more tractable, we further reduced the redundancy in our *G. oxydans* knockout collection, which decreased its size by 85%, and increased its completeness from 93.5% to 100%^81^ by adding additional mutants.

**Fig. 5:**
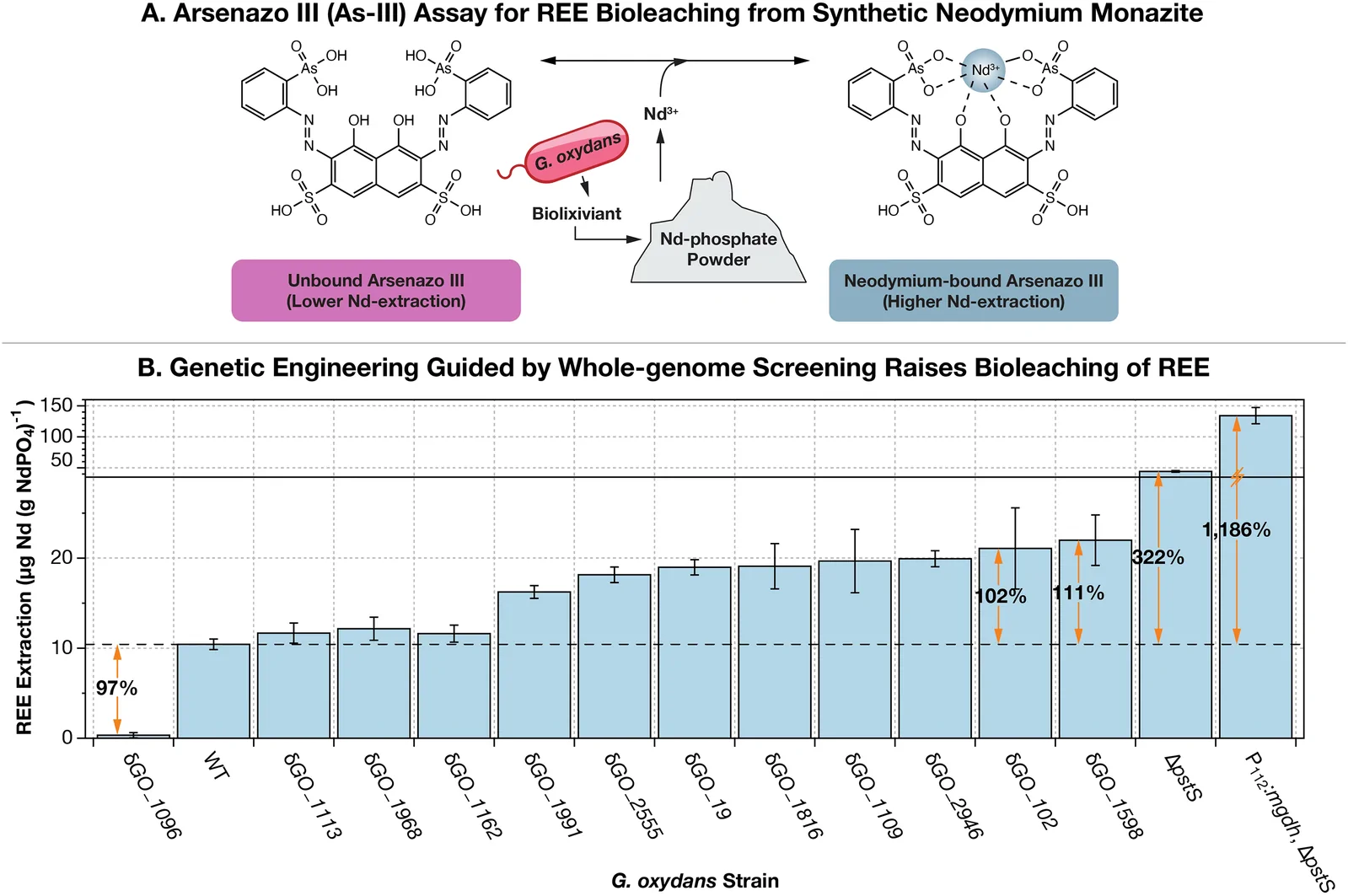
Whole genome screening identifies additional genes that control dissolution of the synthetic neodymium phosphate (colloquially known as synthetic neodymium monazite) that do not affect pH.^82^ (A) We, and other experimenters, have noted that the *G. oxydans* biolixiviant can be much more effective than just gluconic acid (the primary component of the biolixiviant).^78,122,195^ To identify genes that enable this increased effectiveness, we employed a high-throughput screen that uses the lanthanide-chelating dye Arsenazo-III to detect neodymium released by the dissolution of a chemically synthesized neodymium phosphate powder (colloquially known as synthetic monazite) rather than just increased production of acid. (B) *G. oxydans* genes that affect extraction of neodymium from synthetic monazite that do not appreciably impact pH. Note that the *G. oxydans* P_112_:*mgdh*, Δ*pstS* mutant increases extraction from monazite by almost 1200%.^81,82^

Our neodymium phosphate bioleaching screen discovered 68 genes that affected bioleaching but were not previously associated with this mechanism.^81^ Out of the 68, notable changes in pH were observed for only 3 gene disruption mutants, suggesting an important role for non-acid mechanisms in bioleaching. The precise mechanism of these genes remains unclear. Out of the twelve most impactful gene knockouts, 11 increased bioleaching, suggesting that they are regulatory genes, and that the knockout de-represses non-acid bioleaching mechanisms. Knocking out only one gene (*GO_1096*) lowered extraction, reducing bioleaching by 95%, but changed biolixiviant pH by only 0.2 units (in contrast, the knockout of *pqq* genes reduced bioleaching by a similar amount, but increased pH by almost 3 units) (Fig. 5B). Meanwhile, up-regulation of *GO_1096* raised bioleaching by 64%.^81^

What is happening here? The lack of a silver bullet chelator suggests that either *G. oxydans* produces lots of different chelators, none of which on its own has a big impact, or that production of a single chelator is highly redundant. This stands in contrast to acid production, where only a single gene knockout can bring acid production and bioleaching to an almost complete halt.^80^ How these non-acid mechanisms work, and why they seem to be regulated so differently from acid production, remains an outstanding mystery.

### 2.3. Genetically engineered microbes for REE separation

Extracting REEs from rocks and end-of-life feedstocks is only the first step in producing high-purity REEs for industrial applications. But the separation of individual REEs is considered one of the hardest problems in chemistry. The majority of REE chemical separations have focused on the use of commercially available organic solvents and extractants.^91^ These processes give sustainable energy technologies reliant on REE a high environmental and carbon footprint. Because of this, even though mining of REEs has resumed in the United States, only three REE purification plants exist outside of China.^75,92–95^ As a result, new biological and chemical methods^96–99^ have recently been developed to address the challenges of total REE separation,^58,100–105^ light *vs.* heavy REE separation,^60,61^ and individual REE separation.^62,106,107^

Even with unmodified microorganisms, biosorption and desorption from the surface of a cell offered an environmentally friendly route for individual REE separation that was, under a limited set of conditions, already better than solvent extraction.^62^ But, despite promising nascent capability, biosorption was a poorly understood process^100,108,109^ with no obvious roadmap for genetic engineering to make it leapfrog existing technologies.

Our lab completed the first ever comprehensive profile of the genetics of REE biosorption in *Shewanella oneidensis* MR-1. We discovered 242 genes that control the overall level of REE biosorption.^110^ Out of these, 9 genes (most involved in the synthesis of the lipopolysaccharide layer that coats the exterior of *S. oneidensis*) significantly changed REE-binding selectivity by 1 to 4%. This does not sound like much, but modeling suggests that these changes could already reduce the length of the repeated enrichment process by up to 27%.^110^

This inspired us to take a deeper look at this process and ask how much genetic engineering we would need to do to really improve REE separation. In a class-based design study (Cornell Biological and Environmental Engineering 3280) we found that larger multi-locus gene edits (editing 3 or 4 of the 9 genes identified above) could reduce the length of a separation process by almost 90%.^111^

But, *S. oneidensis* is difficult to engineer, and achieving the high performance predicted by genetic engineering would be a challenge. So, we switched to the extremely fast-growing, highly engineerable microbe *Vibrio natriegens*. We used multiple rounds of *in vivo* random mutagenesis to improve its capacity for biosorbing REEs by 210% (Fig. 6A) and to enhance its selectivity between the lightest (lanthanum) and heaviest (lutetium) REEs by up to 50%, and that between adjacent heavy REEs (thulium and ytterbium) by 15%^112^ (Fig. 6B). We think that this last breakthrough allows biosorption to leapfrog small molecule separations for adjacent heavy REEs,^112^ and potentially reduces the length of a separation process by almost 50% (Fig. 6C).

**Fig. 6:**
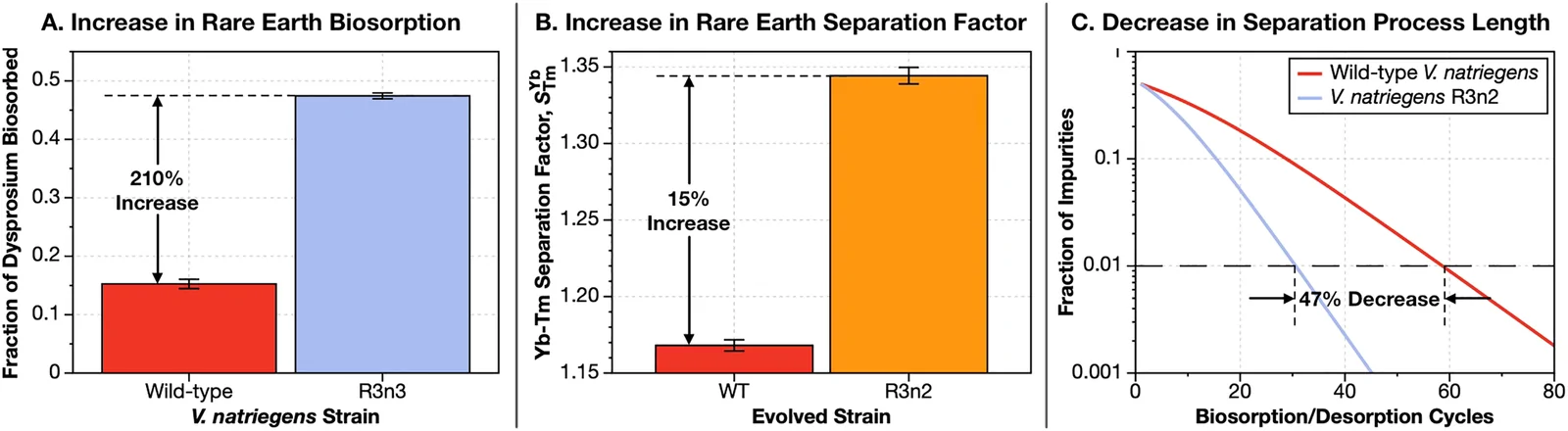
We used whole genome screening of *Shewanella oneidensis*,^110^ and then directed evolution of *Vibrio natriegens*^112^ to improve total REE-biosorption and its selectivity. (A) Three rounds of whole-genome directed evolution of *V. natriegens* improved total biosorption of dysprosium by 210%^112^ over the wild-type (WT). (B) These three rounds of directed evolution improved the separation factor between ytterbium and thulium (two adjacent heavy REE) by up to 15%.^112^ (C) This theoretically reduced the length of a separation process by 47%,^111,112^ potentially leap-frogging conventional solvent extractions.^62^

### 2.4. Ultramafic bioleaching and accelerated weathering

Even when global society achieves net-zero CO_2_ emissions, we will need to remove 10 to 20 billion tonnes of CO_2_ from the atmosphere and oceans every year for decades to prevent warming above 1.5 °C.^113^ There is a surprising amount of uncertainty on the amount of excess CO_2_ in the atmosphere, with estimates ranging from 1 trillion tonnes,^114^ to an often-quoted estimate of 1.5 trillion tonnes,^115,116^ with a recent high estimate of 1.73 trillion tonnes.^117^ Nature permanently removes atmospheric CO_2_ through mineralization, where ultramafic rocks slowly weather, releasing Mg^2+^, Ca^2+^, and Fe^2+^, which react with CO_2_ to form carbonates.^118^ But, this process is much too slow to solve the climate challenge on time,^119^ extending into tens or even hundreds of thousands of years to remove the excess CO_2_ from the atmosphere caused by anthropogenic emissions.^120^ In order to prevent damaging warming of the atmosphere, this problem needs to be solved in approximately 100 years.^113^

In 2019, one of my first MEng students (Rae Brigham) proposed that we could use bioleaching to accelerate weathering of ultramafic rocks,^121^ release Mg^2+^ and Fe^2+^ for CO_2_ sequestration, and make metals like Ni and Co available for sustainable energy technologies. We estimate that the surface-accessible ultramafic material could sequester up to 58× the excess CO_2_ in the atmosphere.^122^ On top of this, it is estimated that there are approximately 10 gigatonnes of ultramafic mine tailings across the globe^118^ that could sequester up to 4.8 gigatonnes of CO_2_.^123^ An additional 400 megatonnes of ultramafic mine tailings are produced every year^118^ that can sequester up to 192 megatonnes of CO_2_. It is important to remember that large-scale CO_2_ removal (tens of gigatonnes per year) does not need to go into operation until approximately 2070, so while these numbers are approximately two orders of magnitude smaller than the total and annual amount of CO_2_ that needs to be removed from the atmosphere, they offer a useful place to get started immediately.

However, our experience of biomining REEs told us that this approach might require an enormous amount of sugar. From everyday experience we tend to think of sugar as a commodity with almost limitless supply, but in reality this supply is finite and could be exhausted by industrial bioprocesses.^124^ We performed a design study on CO_2_ sequestration accelerated by bioleaching to address the sugar demand^125^ and discovered that if the bioleaching was highly efficient (but still within the bounds of realistic), then we could capture tens of gigatonnes of CO_2_ and still rely on sugar to power the bioleaching microbe. However, if multiple aspects of the bioleaching process were even slightly less than ideal, then this carbon sequestration process could easily monopolize the world's sugar and biomass supply, forcing uncomfortable decisions on society like loss of wilderness, mass adoption of vegetarian diets or even population control.^124^

In 2023, we started experimental studies of bio-accelerated weathering. Our first priority was to determine just how well (if at all) *G. oxydans* could dissolve ultramafic material. We determined that *G. oxydans* was the best choice amongst a panel of microbes and fungi (including *Sphingomonas desiccabilis* and *Penicillium simplicissimum*) that were suspected to bioleach ultramafic rocks^123^ (Fig. 7A and B). Next, we determined that it could release Ni, Co, and other valuable energy-critical metals whose sale could be used to offset the cost of carbon sequestration.^122,123^ Furthermore, the biolixiviant produced by *G. oxydans* when fed sugars produced by hydrolysis of cellulosic biomass is almost as effective as that when fed glucose, allowing us to access a much more abundant sugar source^122,123^ (Fig. 7C). Most surprisingly, the effectiveness of the *G. oxydans* biolixiviant relative to gluconic acid went up as the ratio of rock to liquid (the pulp density) increased (Fig. 8A–C). In other words, counter to normal expectations, as the experimental conditions became more industrially relevant, the effectiveness of *G. oxydans* increased.

**Fig. 7:**
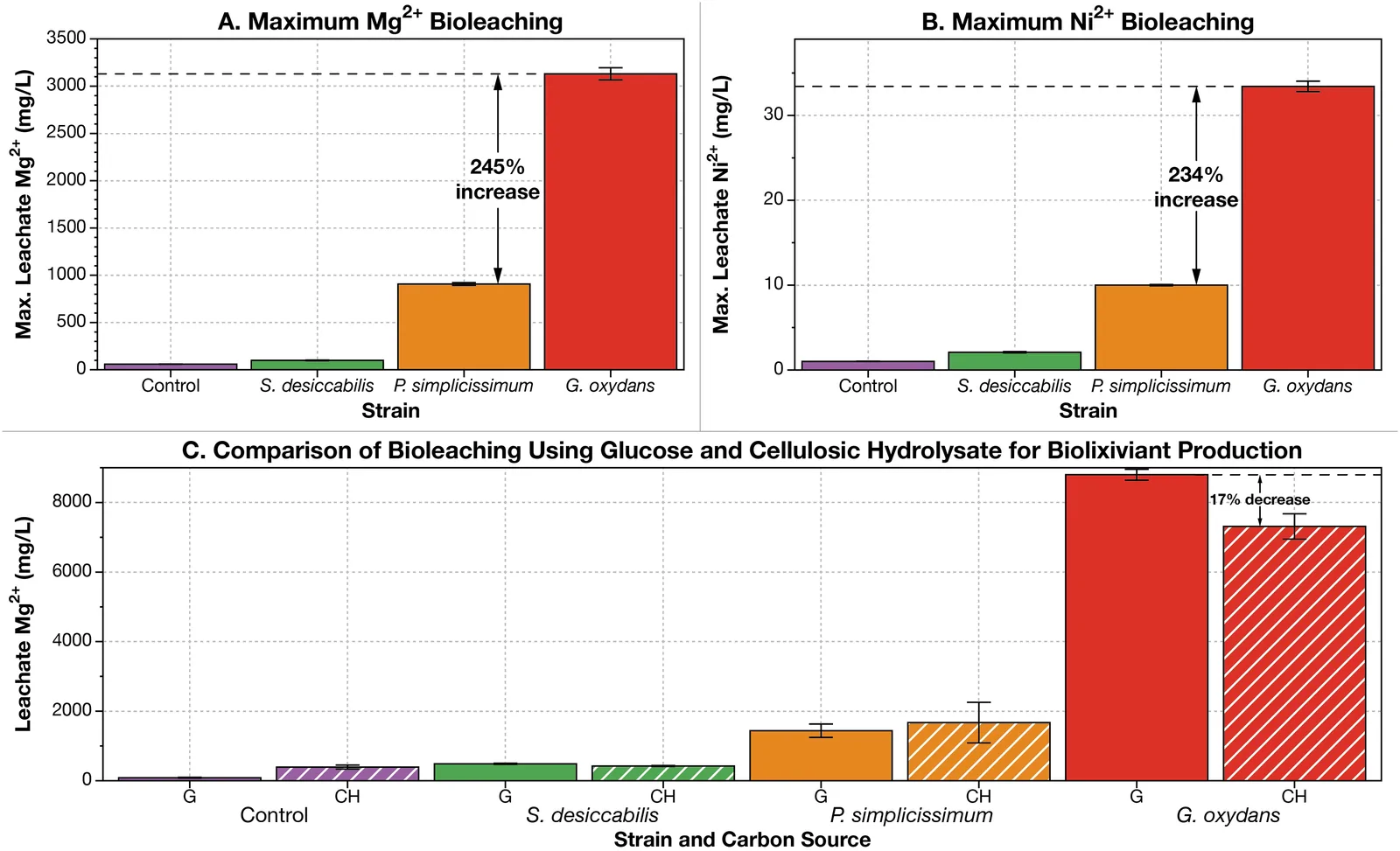
*G. oxydans* is the most effective microbe we have tested for weathering of ultramafic rocks, and can effectively use highly abundant cellulosic feedstocks for biolixiviant production. (A and B) *G. oxydans* produced the highest concentration of Mg^2+^ and Ni^2+^ from the ultramafic rock dunite, followed by *P. simplicissimum* and *S. desiccabilis*. *G. oxydans* produced a maximum Mg^2+^ concentration of 3130 mg L^−1^ after 5 days of leaching at 3% dunite, over 245% higher than the maximum leaching produced by *P. simplicissimum*.^123^ (C) *G. oxydans* and *P. simplicissimum* each successfully bioleached magnesium from dunite using cellulosic hydrolysate instead of glucose. The performance of the *G. oxydans* biolixiviant produced with cellulosic hydrolysate was only 17% lower compared to that of a biolixiviant produced with glucose.^123^

**Fig. 8:**
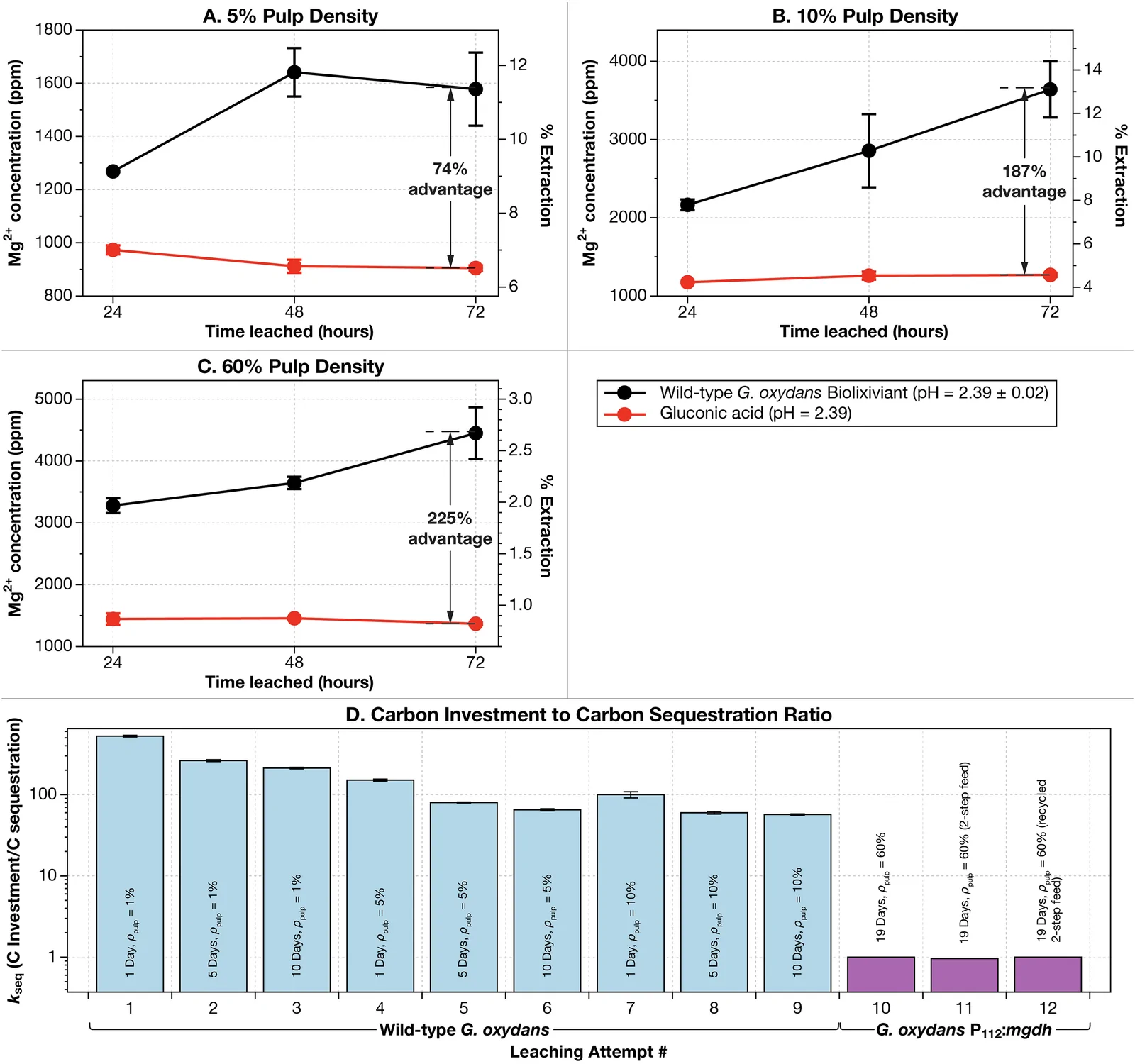
The effectiveness of *G. oxydans* bioleaching of ultramafic rocks increases under increasingly industrially relevant conditions, and we have used this to significantly reduce the amount of feedstock needed to sequester one molecule of CO_2_. (A–C) At 5% pulp density the wild-type *G. oxydan*s biolixiviant is only 1.7× more effective at bioleaching dunite than gluconic acid diluted to the same pH with pure water, but this increased to 3.2× more effective at 60% pulp density. (D) Increasing leaching time and pulp density while reducing average particle size decreased the glucose carbon investment *vs.* carbon sequestration ratio (*k*_seq_) by over two orders of magnitude. Increasing dunite pulp density from 1% to 10% had a larger impact in reducing *k*_seq_ than increasing leaching time from 1 to 10 days. Combining the *G. oxydans* Δ*pstS*, P_112_:*mdgh* double mutant with high pulp densities and long leaching times resulted in a *k*_seq_ value of 1 in three experiments (Col. 10–12). Leaching attempts #1–9 used a dunite sample containing 64% olivine, and #10–12 used a dunite sample containing 92% olivine with a particle size of less than 74 microns as the leaching substrate.^122^

We measure progress in bio-accelerated weathering by the ratio of the number of carbon atoms in the feedstock (*e.g.*, glucose) needed to liberate one magnesium ion (and hence sequester the single atom of carbon in a CO_2_ molecule (*k*_seq_))^122^ (Fig. 8D). We have been able to reduce *k*_seq_ from 525 all the way down to 1 through genetic and process improvements.^122^ If we could reduce *k*_seq_ to 1 at industrial scale, the cost of carbon sequestration (at least the cost of glucose) through bio-accelerated weathering starts to become comparable to (though still expensive) current technologies, at $682 per tonne.^126^ If we could reduce *k*_seq_ to 0.15 (about a 6-fold reduction over our best attempt, but much smaller than the reduction we have already achieved) then we could reduce the feedstock cost for sequestering 1 tonne of CO_2_ to only $100, the target set by the US Department of Energy.^127^ This would not account for the costs of mining ultramafic material, nor does it account for the offset cost of energy-critical metal recovery. However, using this to sequester 20 gigatonnes of CO_2_ a year^113^ would require 2 billion tonnes of glucose, over 10-times current global annual sugar production.^128^

Sugars derived from cellulosic biomass are potentially much more abundant than glucose that is derived from cereal crops like corn, sugar cane, and sugar beet.^129^ While there is no widespread production of cellulosic sugars today, this could happen in the future. Furthermore, it is estimated that cellulosic sugar production could be as low as $100 per tonne^130^ (in contrast to around $1,000 per tonne for glucose). Although cellulosic sugars are abundant, they are not limitless. Slade *et al.* estimated that the impact on the biosphere would be manageable if biomass was withdrawn at a rate of less than ≈7 gigatonnes per year.^124^ We estimate that in order to reduce the cellulosic biomass demand for sequestering 20 gigatonnes of CO_2_ to 3.5 gigatonnes (giving us a safety margin of 50%), *k*_seq_ needs only to be reduced to 0.25. Upcoming techno-economic and life cycle analyses of bio-accelerated weathering from our lab will examine this issue in much greater detail.

How can we further improve the carbon efficiency of biomining? We believe that part of the answer to this problem lies in much better understanding of how microbes interact with metals and minerals. In the past year, our lab, along with those of Esteban Gazel, Matthew Schrenk, Christopher Mason, and Sarah Kreps, started the Microbe-Mineral Atlas Center to uncover these mechanisms and harness them for biomining metals for sustainable technologies and accelerated weathering.

Even if we do harness all of highly efficient mechanisms that nature offers for the transformation of metals and minerals, we recognize that the availability and cost of carbohydrate feedstocks will likely remain a challenge. Right now, sugars are often the limiting factor in the economic viability of industrial bioprocesses. Could there be a better, cheaper way to make them?

## 3. Electromicrobial production and hyper-engineerable chassis organisms

### 3.1. Introduction to electromicrobial production

In the near future, it is almost inevitable that carbon-free renewable electricity will become abundant.^131^ Unfortunately, not every technology can be easily electrified, including aviation, shipping, and chemical and food production. While many of the molecules we need can come from photosynthesis, its low efficiency^132,133^ poses an enormous challenge.^134^ Conversion of electricity into valuable chemicals presents a big opportunity.

Electromicrobial production (EMP) is a class of technologies that use renewable electricity to power microbial metabolism^135–138^ to reduce CO_2_ or simple electrochemically reduced forms of CO_2_ like formate (HCOO^−^) into complex, highly reduced molecules like biofuels, lubricants, foods, polymer precursors, biolixiviants for biomining, and sugars. If electricity is supplied by photovoltaics, then the sunlight-to-chemical conversion efficiency might exceed that of photosynthesis.^134,139^

There are at least three ways to introduce electricity into a metabolic system: (1) oxidation of electrochemically produced hydrogen^140^ (Fig. 9A); (2) oxidation of electrochemically reduced CO_2_ compounds like formate, carbon monoxide and methanol (all C_1_)^137,141,142^ (Fig. 9B); and (3) direct uptake of electrons from soluble mediators, direct contact with an electrode, or by long-range electron transfer through microbial nanowires produced by electroactive microbes in a process called extracellular electron uptake (EEU)^143^ (Fig. 9C).

**Fig. 9:**
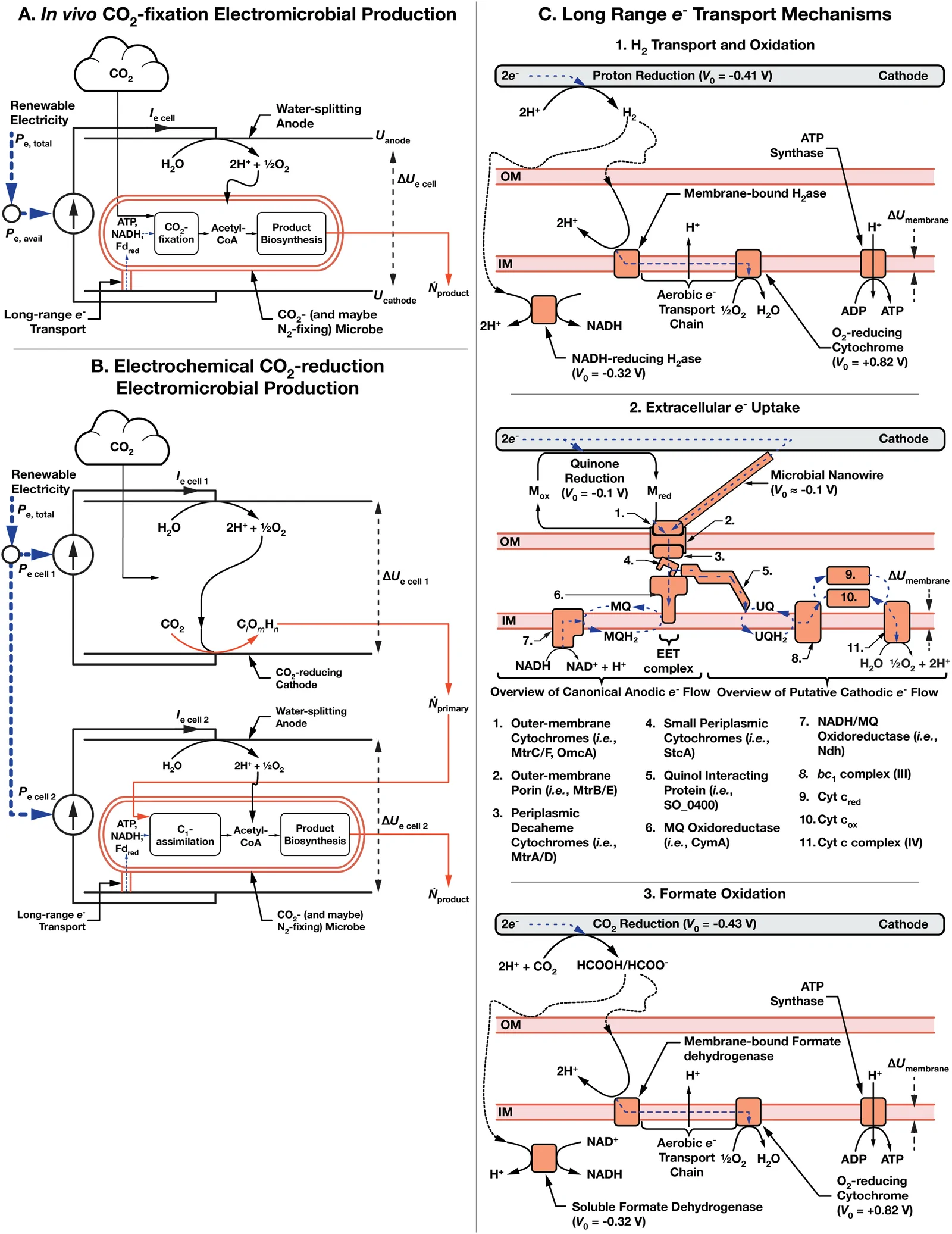
Overview of electromicrobial production technologies. (A) A microbe absorbs electrical power, *P*_e,avail_, through H_2_-oxidation, oxidation of an electrochemically reduced C_1_ compound like formate, or through direct extracellular electron uptake (EEU) to power CO_2_-fixation and bioproduct production at a rate *Ṅ*_product_. The total electrical power is used to drive a current, *I*_e cell_, across a whole-cell voltage, Δ*U*_e cell_, and can also be used to power an agitator. (B) The electrical power is split between two electrochemical cells. In the first, CO_2_ is reduced to a short chain hydrocarbon like formate at a rate *Ṅ*_primary_. The primary fixation product is then concatenated and further reduced in the second cell by a H_2_-oxidizing, C_1_-oxidizing, or electroactive microbe. Electrons are transported to metabolism by either (C.1) diffusion or stirring of H_2_ and oxidation by a hydrogenase (H_2_ase) enzyme; (C.2) diffusion or stirring of a soluble mediator like a quinone or directly across a conductive extracellular matrix (ECM) and transfer into an electroactive cell by a membrane-spanning extracellular electron transfer (EET) complex, or (C.3) diffusion or stirring of formate and oxidation by formate dehydrogenase. This figure was adapted from ref. 170 under the terms of a Creative Commons Attribution 4.0 International License.

I first started work on EMP as a postdoctoral fellow at Harvard Medical School. All methods of electron delivery have unique advantages and disadvantages. While H_2_-mediated EMP is the easiest to start and gain traction due to the genetic engineerability of H_2_-oxidizing, CO_2_-fixing microbes like *Cupriavidus necator* (historically known as *Ralstonia eutropha*),^140,144^ I intuited that it would face challenges during the scale-up due to the low solubility of H_2_ in water (*i.e.*, the energy demands of transferring H_2_ into water would significantly reduce energy conversion efficiency).^145^ Meanwhile, I was concerned that the dual use of electrochemically reduced C_1_ compounds as both carbon and electron donors could introduce energy efficiency losses.

On the other hand, I hypothesized that the challenges faced by EEU-mediated EMP were rooted in scientific uncertainty and the poor genetic engineerability of electroactive organisms known at the time (2011) like *Shewanella oneidensis*. If I could bridge this knowledge gap and build new advanced genetic engineering tools, EEU would have the significant potential to scale-up and make a difference in the real world.

As an independent fellow at Princeton and later as a faculty member at Cornell, my lab has made significant discoveries that reduce the scientific and technical uncertainty and risk around EEU-mediated EMP and laid a foundation for creation of a highly scalable system to convert CO_2_ and electricity to valuable chemicals with high efficiency in the coming years.

### 3.2. Outstanding scientific and engineering challenges of EEU-mediated EMP

Despite my enthusiasm for EEU-mediated EMP, it faced at least two major challenges that worked together to paralyze efforts to exploit it. First, when I started this work as a postdoctoral fellow, there was no known microbe that was capable of EEU, capable of CO_2_-fixation or assimilation of electrochemically reduced C_1_ compounds, and that was easy to genetically engineer so it could be tailored to the demands of an industrial process.

For example, the model electroactive microbe *S. oneidensis* is genetically engineerable (although with more difficulty than the synthetic biology workhorse *E. coli*). But, unlike *C. necator* (a H_2_-oxidizing microbe), *S. oneidensis* was not known to naturally fix CO_2_. This means that it would have to be genetically engineered with genes encoding a CO_2_-fixation cycle. At the time of starting this work, no one had achieved anything close to this feat. As an aside, recently it has been discovered that with some small genetic edits, a naturally occurring CO_2_-fixation pathway, the reductive glycine pathway,^146^ can be activated in *S. oneidensis*.^147^

Second, the energetics of the EEU process were a black box. *S. oneidensis* is well-known for transferring electrons from its metabolism to solid substrates like minerals and even electrodes in a process called extracellular electron transfer (EET).^148,149^ This is enabled by a special protein complex called Mtr that connects the electron transport chain in its inner membrane to the exterior of the cell. At first glance, it looked like this process could be reversed and *S. oneidensis* could uptake electrons to power metabolism.^150^ But, the Mtr EET complex accepts electrons at a lower energy than that of NADH (the primary reductant used in CO_2_ fixation), so they cannot directly reduce it.^151^

In order for EEU to power CO_2_-fixation, it seems likely that some sort of electron energy up-conversion has to happen, in a process called “the uphill pathway”.^152^ Again, this is in stark contrast with H_2_-mediated EMP where the redox potential of H_2_ is sufficiently low (−0.42 V *vs.* the standard hydrogen electrode (SHE)) that it can directly reduce NADH (−0.32 V *vs.* SHE).^135^ While some microbes certainly possess this machinery,^153,154^ it was unclear if *S. oneidensis* was one of them.

Even if *S. oneidensis* did possess the EEU machinery, would it be efficient enough? This is especially relevant given that the measured energy conversion efficiency of an H_2_-mediated EMP system powered by a solar photovoltaic using *C. necator* already exceeds the theoretical maximum efficiency of photosynthesis by a wide margin.^140^ This was, and remains, a high bar for *S. oneidensis* to clear. Despite the uncertainty around EEU, I still felt that it offered the best way to build a highly scalable EMP system. So, I needed to get right to the heart of the matter and find out if *S. oneidensis* could uptake electrons, how the process worked, and make an estimate of its upper-limit efficiency.

### 3.3. Development of Knockout Sudoku for characterizing genomes of non-model microbes

The most obvious way to answer if *S. oneidensis* is capable of EEU was to directly measure it in a bio-electrochemical system. However, this seemed daunting, and was not achieved until 2018, 7 years later.^143^ Rather than wait 7 years, I decided to tackle the problem with a different approach: a genetic screen to identify the genes that *S. oneidensis* uses for electron uptake.

Genetic screening requires two things: (1) an assay for the phenotype under study (*i.e.*, electron uptake), and (2) a collection of gene knockout mutants. Inspired by Dianne Newman's work,^155^ I built an assay for EEU that measures oxidation of the redox dye anthrahydroquinone-2,6-disulfonate (AHDS_red_, the reduced form of anthraquinone-2,6-disulfonate or AQDS_ox_).

The most accessible technology for building a mutant collection is transposon mutagenesis.^156,157^ Unfortunately, conventional transposon mutagenesis collections leave a lot to be desired. First, we know that if the collection is big enough it will probably contain a mutant for every non-essential gene in the organism's genome.^158^ However, we will not know exactly where the transposon is in each gene and what chance it has of disrupting gene function, nor where each mutant is in the transposon collection. Michael Baym and I calculated that for a genome of *S. oneidensis*' size (≈3700 non-essential genes) I would need to pick at least 24 000 mutants to cover all of its genes.^87^ The biggest transposon mutant collection most experimenters can handle has only about 5000 to 8000 members, meaning that many genes are not covered by the screen.

Second, because we do not know where any mutant is in a random transposon collection, we cannot test specific gene function predictions.^159^ For example, I predicted that disrupting the *mtr* genes that code for the *S. oneidensis* EET complex should eliminate or slow AHDS_red_ oxidation, but without knowing where these mutants were, there was no way to test this prediction.

Third, we could not confirm that the transposon collection was fully random. For example, although we took every precaution to prevent *S. oneidensis* replication during preparation of the disruption library, this could not be guaranteed. I noted that disruptions of the *pta* gene appeared repeatedly in my screen results, but could not determine whether this reflected true biological importance of this gene in electron uptake, or over-representation of *pta* knockouts in the initial library due to a differential fitness advantage.

I realized that I needed a whole genome knockout collection of *S. oneidensis*. This is a comprehensive set of single-gene knockout mutants (one for each of thousands of non-essential genes in an organism's genome) that have been clonally isolated, catalogued and arrayed onto microwell – typically 96-well – plates. The usefulness of these collections is hard to overstate: in the 15 years since the release of the gold standard Yeast Knockout Collection of *Saccharomyces cerevisiae* (baker's yeast), it was used in over 1000 genome-wide experimental screens.^160^

The Yeast Knockout Collection cost ≈$2 million and 4 years to build (when adjusted for inflation this is ≈$4 million).^160^ The gold standard Keio collection for *E. coli* likely had a similar construction time and cost.^161^ More recent methods can reduce costs enormously, but are still prohibitive^87^ at hundreds of thousands of dollars.

I had neither the time nor the money for state-of-the-art mutant collection construction techniques for *S. oneidensis* – I needed to come up with a method for building a knockout collection that was faster and cheaper than anything that preceded it. At this point in 2014, I left my postdoc at Harvard Medical School and moved to an independent fellow position in the Chemistry Department at Princeton.

As soon as I got to Princeton I set about building the knockout collection for *S. oneidensis*. I realized that I could afford a Cartesian pooling scheme where aliquots from each mutant are dispatched to four pools determined by the row and column, and plate-row and plate-column coordinates of the mutant. This scheme can be performed with only a 96-channel pipettor and a handful of specialty row and column micro-plates.^87^

A few years prior, Yaniv Erlich had predicted that this simple scheme would fail because identical transposon mutants can appear multiple times.^162^ For example, if a mutant appears two times (very likely in a truly random collection), this will result in eight coordinates, which can be recombined in 2^4^ = 16 possible locations, only 2 of which are real. I developed the Knockout Sudoku algorithm that uses probabilistic inference to calculate which location assignments for a mutant are real.^87^ This approach allowed us to catalog ≈80% of the 40 000 mutants in the *S. oneidensis* progenitor collection, and find mutants for ≈99% of all non-essential genes. At the end of all this, instead of spending millions of dollars, we had spent only about $10 000.

The *S. oneidensis* whole genome knockout collection allowed me to re-discover almost all of the genes associated with EET over the previous 16 years in a single experiment.^87^ The stage was now set for discovering genes involved in EEU.

### 3.4. Discovery of a pathway for extracellular electron uptake

We screened the *S. oneidensis* whole genome knockout collection^87,163^ with the AHDS_red_ oxidation screen and discovered 149 genes that were associated with EEU^164^ (Fig. 10). Using a combination of sequence annotation and intuition, I narrowed down the list of genes that I thought were truly important in EEU down to 41. The next challenge was proving that any of these 41 genes were directly involved with EEU. Luckily, Annette Rowe, then a postdoctoral fellow at the University of Southern California, had recently provided compelling evidence using bio-electrochemical measurements that *S. oneidensis* was capable of using EEU to regenerate ATP and NADH,^143^ but she did not know the genetics of the process. We decided to team up. I made a best guess and selected 24 genes (the maximum amount Dr Rowe believed she could process) and sent them to her new lab at the University of Cincinnati for bio-electrochemical analysis.

**Fig. 10:**
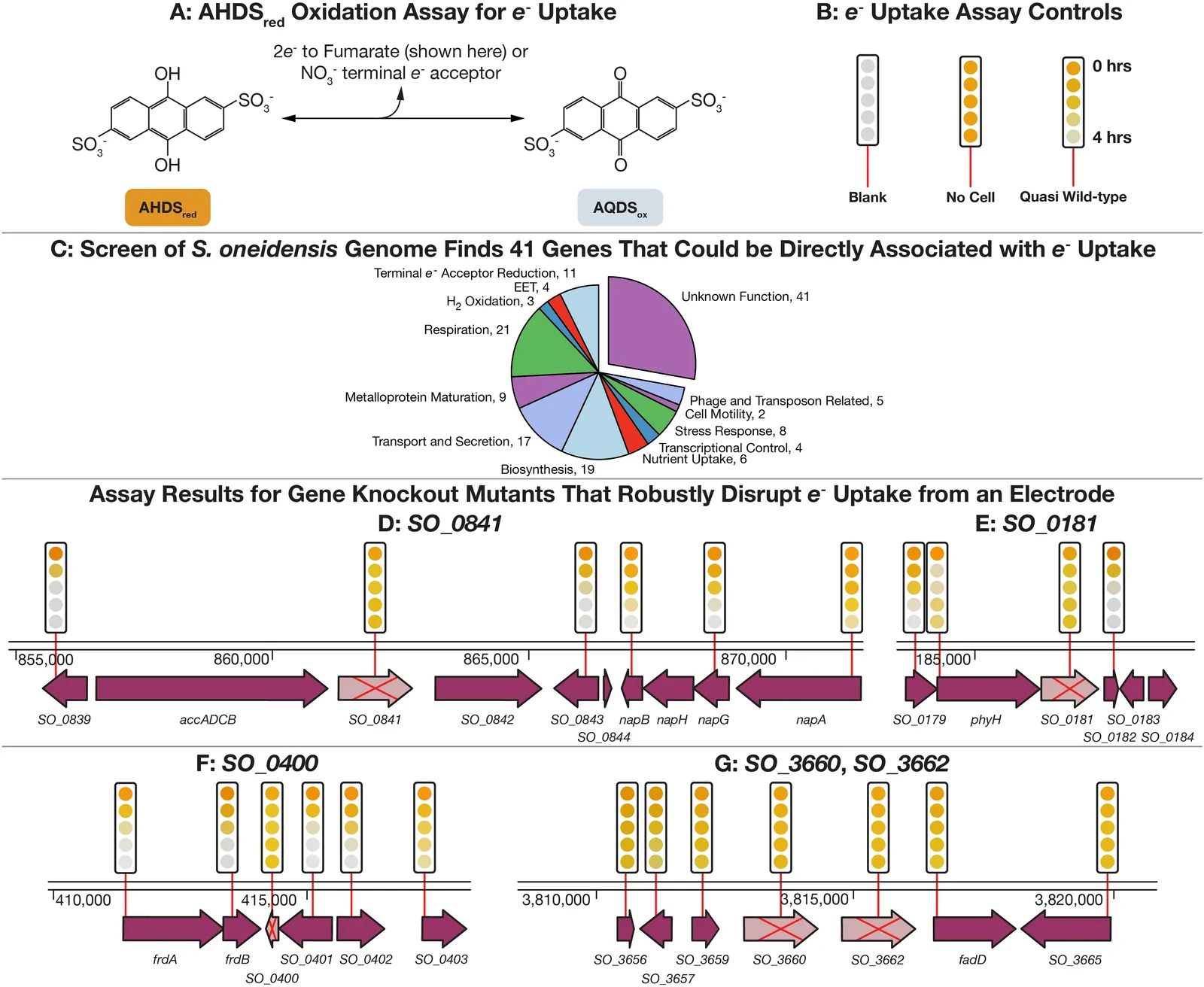
We used whole genome screening of the model electroactive microorganism *Shewanella oneidensis* MR-1 to find 149 genes that disrupt electron uptake. All 3712 members of the *S. oneidensis* whole genome knockout collection^87^ were screened for electron uptake capability with an AHDS_red_ (anthrahydroquinone-2,6-disulfonate, the reduced form of anthraquinone-2,6-disulfonate or AQDS_ox_) oxidation assay. (A) AHDS_red_/AQDS_ox_ redox reaction is used as a proxy for extracellular electron uptake. AHDS_red_ changes color from orange to clear when oxidized. Electrons are transferred to either a fumarate or nitrate terminal electron acceptor by *S. oneidensis*. Data shown use fumarate. (B) Blank, no-cell and quasi-wild-type (transposon mutants that contain a kanamycin cassette but have no effect on AHDS_red_ oxidation) controls. The color of the AHDS_red_ dye is recorded photographically and displayed at 1 hour intervals after the start of the experiment by a series of colored circles above each gene. (C) The electron uptake assay associates 149 genes with electron uptake. Electron uptake failure can be explained in 108 cases, but in 41 cases it fails for unknown reasons, implicating these genes in a novel electron uptake process. (D–G) AHDS_red_ oxidation assay results for deletion mutants containing knockouts of genes highlighted in this article (pink arrow with a red cross through the center) along with gene disruption mutants for surrounding genes (purple arrow, with a red line indicating the location of the transposon insertion). This figure was reproduced from ref. 196 under the terms of a Creative Commons Attribution 4.0 International License.

In total, Dr Rowe's group found that 5 of the 24 gene knockout mutants were indispensable for EEU from a cathode. On top of this, in 4 of 5 cases (*SO_0181*, *SO_0400*, *SO_3660*, and *SO_3662*) these gene knockouts produced no significant defect in electron donation to an anode. Only knockout of *SO_0841* reduces anode donation as well. We believe that these genes enable the “uphill pathway” by connecting aerobic and anaerobic electron transport chains of *S. oneidensis* (Fig. 11). Homologs of these genes are found across genera, which suggests that electron uptake could be widespread.^165^ These discoveries provide a foundation for studying electron uptake in exotic metal-oxidizing autotrophic microbes, genetic improvement of electron uptake, and engineering electron uptake into a highly tractable host like *E. coli* or *Vibrio natriegens* to complement recent advances in synthetic CO_2_ fixation.

**Fig. 11:**
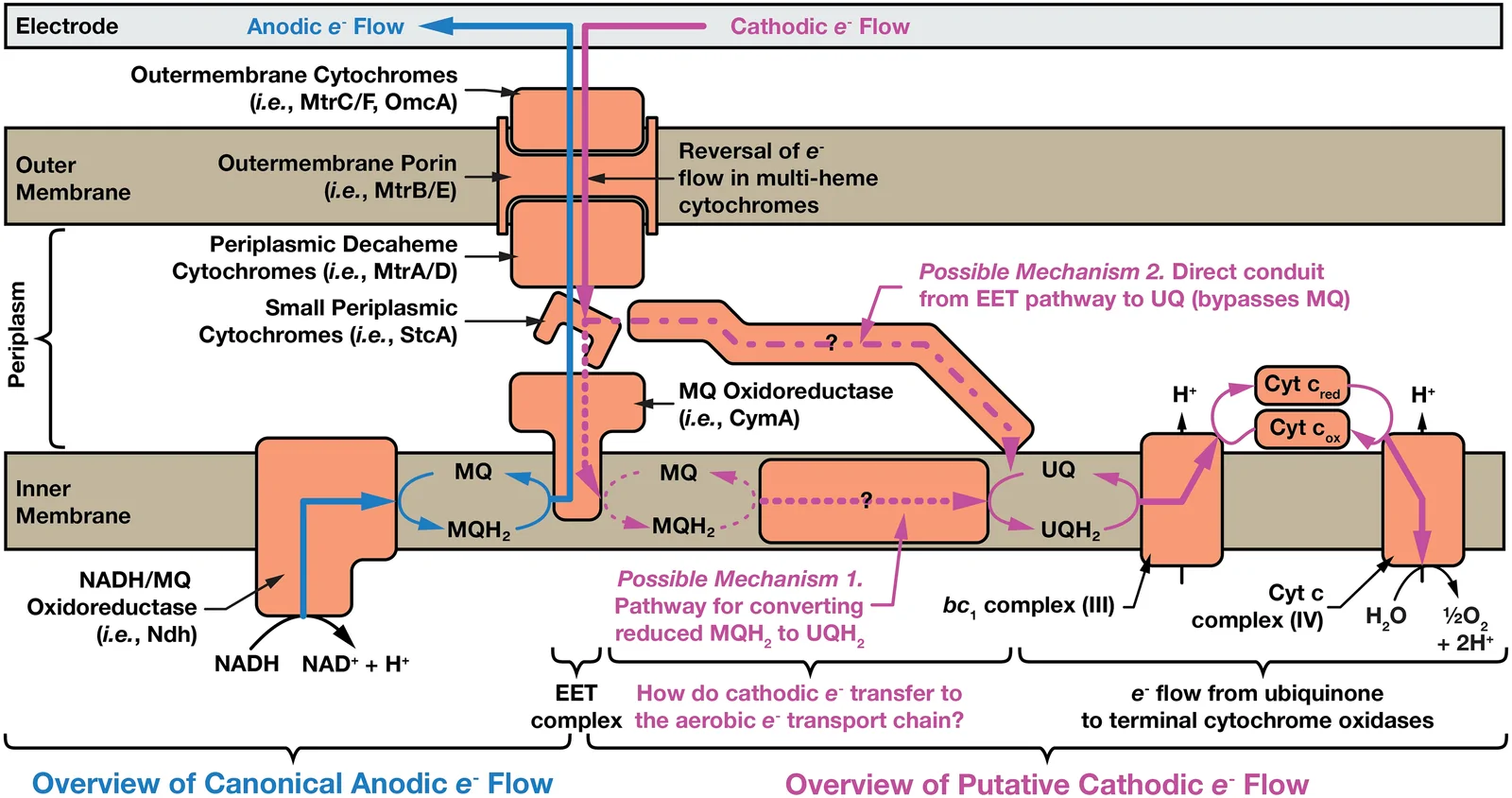
A proposed newly discovered pathway for electron uptake in the model electroactive organism *Shewanella oneidensis* MR-1. *S. oneidensis* was known to be capable of electron uptake, but this could not be fully explained by reversal of the well-characterized extracellular electron transfer (EET) pathway. The canonical EET pathway for electron transfer from the cell interior and anaerobic respiration is shown in light blue. Parts of the electron uptake pathway that were known prior to our work are denoted with solid pink lines. However, there were two possible mechanisms for transfer of cathodic electrons from the Mtr EET complex to the ubiquinone pool and onto terminal cytochrome oxidases that are shown as dashed lines. Electrochemical measurements by Annette Rowe's lab suggest that mechanism 2 is more likely and is formed by two of the proteins identified by genetic screening (SO_0400 and SO_3662) (performed in our lab and shown in Fig. 10). This figure was reproduced from ref. 196 under the terms of a Creative Commons Attribution 4.0 International License.

### 3.5. Theory of electromicrobial production

As I was developing Knockout Sudoku to discover the genes that controlled EEU, to eventually genetically engineer it, I wanted to predict if any of it was worth doing. I set about building a model of EEU to calculate its upper-limit efficiency. This model connected the details of the bio-electrochemical system that hosts the EMP microbes down to molecular details of the electron uptake mechanism, and the metabolism that converts CO_2_ or electrochemically reduced CO_2_ to a biofuel.

My team built a computer code to calculate the efficiency of electromicrobial production of the biofuels butanol and isopropanol, using six naturally occurring CO_2_-fixation pathways, the artificial CETCH CO_2_-fixation pathway,^166^ and the formolase formate-assimilation pathway.^167^

We predicted that the upper-limit efficiencies of H_2_- and EEU-mediated EMP were far higher than any form of photosynthesis.^138^ The next truly surprising result was that the upper-limit efficiency of EEU-mediated EMP, despite all our intuition, was only ≈2–5% lower than H_2_-mediated EMP.^138^ This result was so shocking to me that when I calculated it in 2017, I held off publication for 3 years to check and re-check the calculation. The third major result we found is that we can increase the efficiency of EMP by almost 20% by changing the method of CO_2_-fixation^138^ (Fig. 12).

**Fig. 12:**
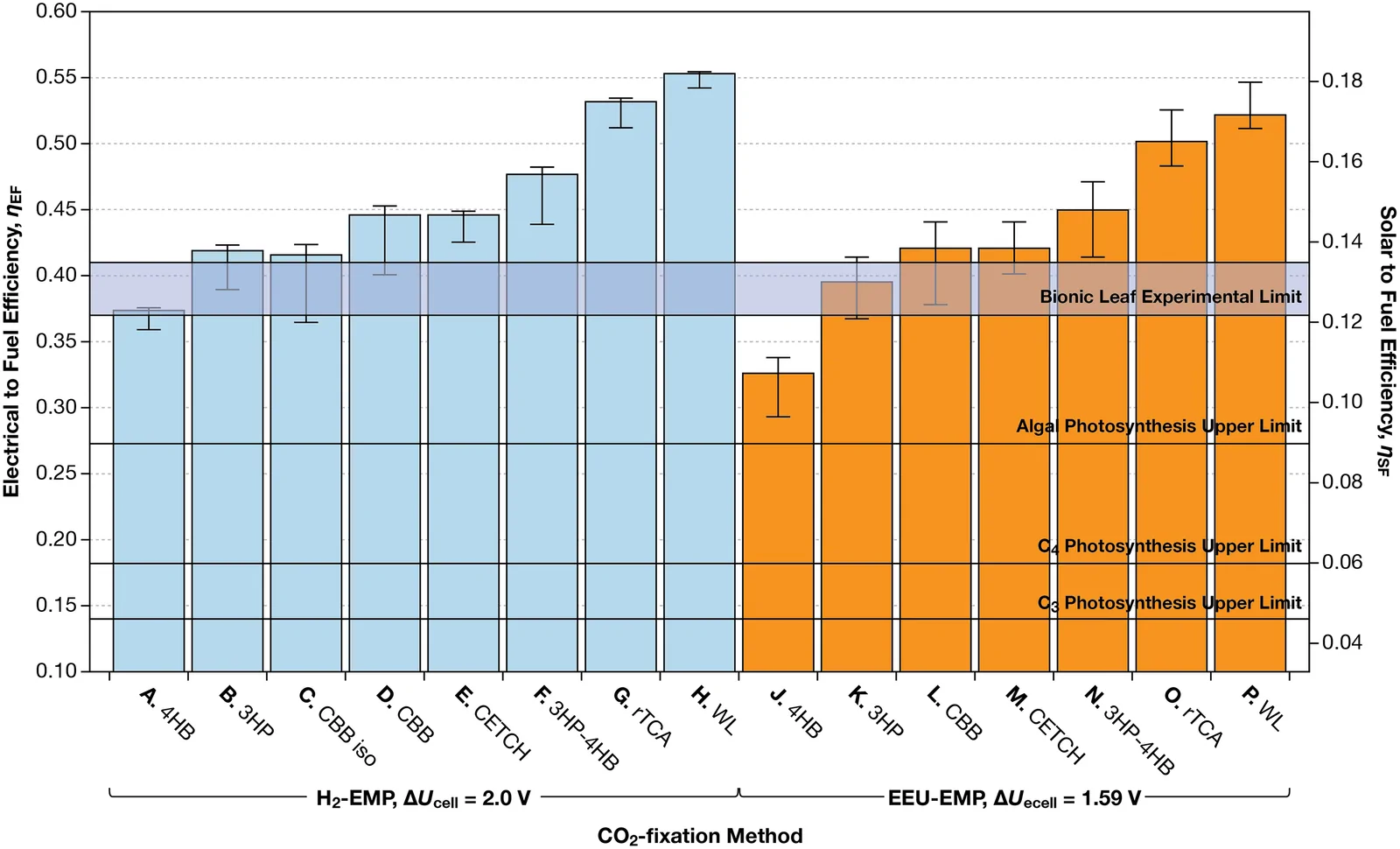
Projected lab-scale electrical and solar to biofuel efficiency of electromicrobial production schemes. The right axis is calculated by assuming a solar to electrical conversion efficiency of 32.9%, the maximum theoretical efficiency of a single junction Si solar PV.^197^ Whole cell voltages were calculated from the minimum redox potentials of H_2_ and the Mtr EET complex^151^ midpoint redox potentials, and from bias voltages reported in ref. 140, 144 and 198. The error bars are due to uncertainty in the trans-inner membrane voltage and span 80 mV (BioNumbers ID (BNID) 104082, see ref. 199) to 270 mV (BNID 107135), with a central value of 140 mV (BNIDs 109774, 103386, and 109775). All efficiencies are for butanol production, except where noted as isopropanol (iso). 4HB = 4-hydroxybutyrate cycle; 3HP = 3-hydroxypropionate bicycle; CBB = Calvin–Benson–Bassham cycle; CETCH = (CoA)/ethylmalonyl-CoA/hydroxybutyryl-CoA; 3HP-4HB = 3-hydroxypropionate 4-hydroxybutyrate bicycle; rTCA = reductive tricarboxylic acid cycle; WL = Wood–Ljungdahl pathway. Redrawn with data from Salimijazi *et al.*^138^

Even though the efficiency of EEU-mediated EMP is close to that of H_2_-mediated EMP, it is still lower and the latter already works (*e.g.*, the bionic leaf^140^). Is there any point in pursuing it, given that there are likely to be formidable genetic engineering challenges in combining EEU with CO_2_ fixation (or C_1_-assimilation)? To answer this, I built a model of the minimum energy requirements of mixing H_2_ into water with the help of Richard Grenville at Philadelphia Mixer. This model validated my intuition that an EEU-mediated EMP system would be much easier to scale up than an H_2_-mediated system due to the low solubility of H_2_ in water.^138^ Despite the considerable interest in it for food production, and the relative ease of genetically engineering H_2_-mediated EMP, this may be the issue preventing a commercial breakout.

Since completing the original theory of EMP, my team has expanded it to calculate production efficiency of increasingly complex molecules. In the fall of 2020, my class studied the electromicrobial production of proteins. We predicted that EMP microbes powered by H_2_-oxidation or EEU could produce amino acids with energy inputs as low as 64 MJ kg^−1^, approximately one order of magnitude lower than any previous estimate of electromicrobial protein production.^168^ Next, we applied our EMP theory to the production of jet fuels. We predicted that, for a perfect replica jet-fuel blend, the electrical-to-fuel energy conversion efficiency was 40%^169,170^ (Fig. 12).

My team's work on the upper-limit efficiency of EMP is important because it gives a roadmap and a target for engineering. In 1961, Shockley and Quiesser made an estimate that the upper limit of the efficiency of solar photovoltaics^171^ was about 30% when the highest reported efficiency of a PV device was ≈4%.^172^ Today, just a little over 60 years later, the technology is beginning to reach full maturity with efficiencies approaching that limit.^173^ While we do not know how to solve all of the engineering challenges of EMP, our work indicates that if they can be solved, the payoff could be significant (Fig. 13).

**Fig. 13:**
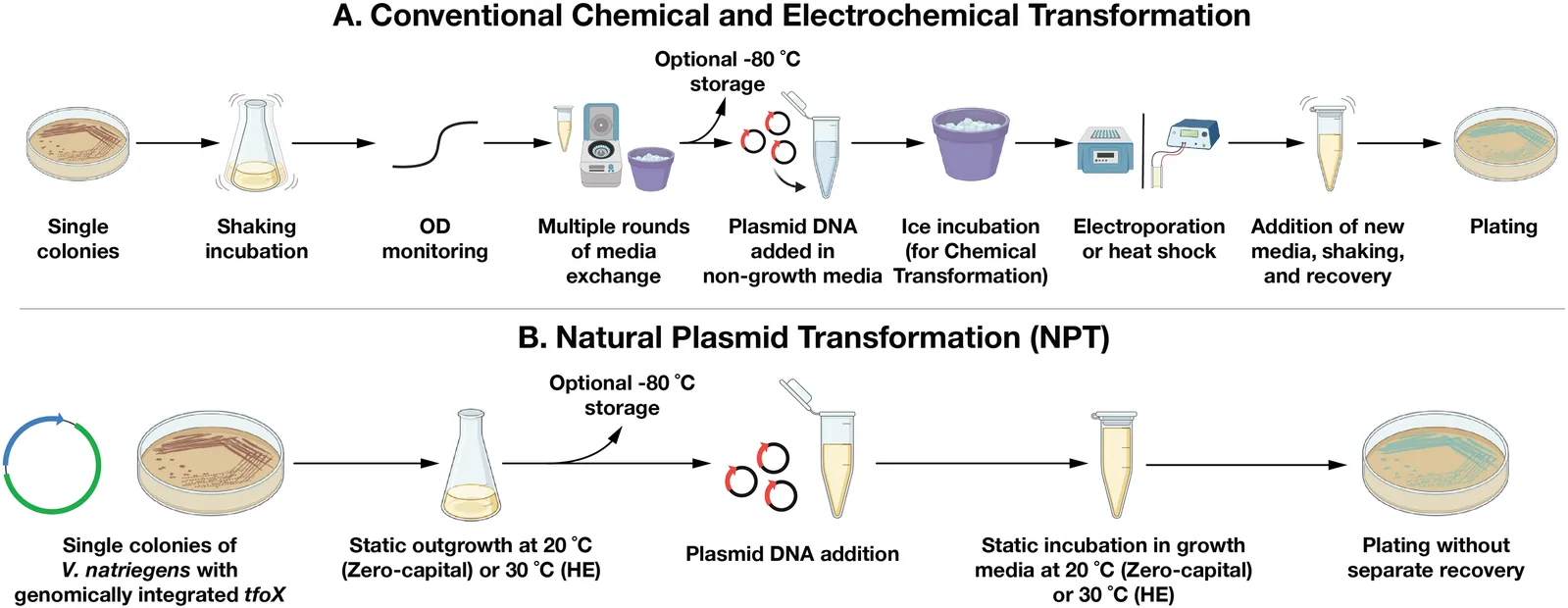
A strain of *Vibrio natriegens* engineered for natural competence is transformable with plasmids directly from competence media without media exchange or addition, electroporation or heat shock, or a separate recovery step. (A) Conventional chemical and electrochemical transformation methods are highly dependent upon expensive capital equipment and hands-on tasks. (B) Natural plasmid transformation is enabled by genomic expression of *tfoX* from *Vibrio cholerae* and allows a much simpler transformation protocol. We have developed two protocols, a high-efficiency protocol (10^6^ colony forming units (cfu) per μg), which requires only the use of an incubator and a deep freezer, and a zero-capital protocol (10^4^ cfu μg^−1^), which requires no capital equipment at all and can be done entirely at room temperature. Because cells are transformed in their growth media, with no further concentration or media exchange, either protocol can be easily scaled, and the high efficiency protocol can be completed with as little as 45 minutes of hands-on time. Redrawn from Specht *et al.*^181^ with BioRender images.

### 3.6. Discovery of fast extracellular electron uptake by H_2_-oxidizing microbes

Despite my concerns regarding H_2_-mediated EMP, it would be foolish to ignore its potential. In collaboration with Peng Chen in Cornell Chemistry, and Tobias Hanrath in Chemical and Biological Engineering, we used single cell and single molecule imaging to measure extracellular electron uptake from light-absorbing semiconductor quantum dots by *Cupriavidus necator*.^174^ The results of these experiments were unexpected.

One of our earliest experiments was to connect *C. necator* cells to cadmium selenide (CdSe) quantum dots. *C. necator* normally acquires electrons for CO_2_-fixation by oxidizing hydrogen, but no H_2_ is produced by CdSe quantum dots, so I was pessimistic about getting results. But we saw that *C. necator* was able to directly uptake about 1000× more electrons than I ever predicted, with a measured current of 1 nanoAmpere.^134^ Upon further investigation, we found that the hydrogenase enzymes that oxidize H_2_ and inject electrons into *C. necator*'s metabolism can also directly uptake electrons from the quantum dot. This result shows that nature can still surprise us, and that we might be able to perform electromicrobial production faster than I ever thought possible.

### 3.7. Accelerated weathering powered by electromicrobial production

As discussed earlier, sugar made from photosynthesis is prohibitively expensive for massive-scale bioprocesses. Could we replace this sugar with sugar made by EMP, or even sidestep sugar entirely by forming the product, *e.g.* biolixiviants, through EMP? We found that EMP allows the biolixiviants needed to sequester a tonne of CO_2_ to be produced for less than $100 (the target set by the US Department of Energy's Carbon Negative Shot Initiative^127^), even with modest bioleaching and CO_2_ mineralization performance.

## 3. Development of a highly engineerable chassis organism

Now that we have established that EEU-mediated EMP could have a high theoretical maximum efficiency, could scale up well, and discovered the unique genes that enable EEU, the next step is to build a highly engineerable organism that combines EEU and CO_2_ fixation.

In principle, building a highly engineerable organism that uptakes electrons and fixes CO_2_ is achievable. The lab workhorse organism *E. coli* has been engineered to fix CO_2_,^175,176^ and to assimilate electrochemical CO_2_ reduction products like formic acid.^177^ Meanwhile, Caroline Ajo-Franklin and coworkers engineered *E. coli* with the *S. oneidensis* Mtr complex to allow it to reduce metals.^178^ Despite this, I predicted that the chances of combining the two systems and achieving our high performance predictions in a reasonable amount of time are low.

My postdoc David Specht and I realized that *E. coli* might not be the right organism to take us forward, and we turned to the extremely fast-growing and engineerable microbe *Vibrio natriegens*.^179^ David and I realized that to combine high-performance EEU and CO_2_-fixation we would likely need to use directed evolution. Whole genome engineering technologies like MAGE (multiplex automated genome engineering)^180^ can dramatically accelerate evolution. But technologies like this are unsuitable for the evolution of an autotrophic (*i.e.*, CO_2_-fixing) metabolism as they rely on high cell densities only achievable in a heterotrophic (*i.e.*, growing on rich media) metabolism.

David and I turned to a phenomenon known as natural competence. Natural competence is a process in which cells use a molecular harpoon to uptake extracellular DNA without heat or electroshock and has been found natively in *V. natriegens*. To improve its performance, we integrated the *tfoX* natural competence master regulator from *Vibrio cholerae* into the genome of *V. natriegens*, and we invented a minimal competence medium (MCM) which supports both growth and a state of natural competence which is maintained for 10s of hours. These breakthroughs enable synthetic biology with no capital equipment at all, allowing synthetic biology to happen in very low resource environments like a high-school or garage biotech startup.^181^ In the next decade, we aim to use naturally competent *V. natriegens* to engineer a high-performing strain capable of electromicrobial production using extracellular electron uptake.

## 4. Limitations and key uncertainties

While we note uncertainties and limitations throughout this article, it is useful to consolidate them and extend their consideration in a single place. The largest assumption in this article is that biology is the right tool for sustainability problems at all. We make the case for this in the Introduction. However, it is useful to consider the opposite position: thermochemistry is not merely entrenched, it is phenomenally successful, and it has won head-to-head contests against biology before.^182^ However, while thermochemistry was undoubtedly the right approach for the problems of the 19th and 20th centuries, biology might be a better approach for the problems of the 21st and 22nd centuries. Chemistry allows processes to reach a scale sufficient to perturb the biosphere. But, operating at a rate that matches or exceeds that of the biosphere might require a different approach. The engineering temptation is to raise biology's rate by putting it in a reactor, and the moment we do that we are faced with many of the same limitations as thermochemistry. Instead, we would like biology to operate at the intensity of thermochemistry, but maintain the cost structure of agriculture. Every configuration we propose can be scored on whether it is closer to biology's cost structure or to chemistry's.

### 4.1. Biomining and bio-accelerated weathering

Copper bioleaching, taken as the existence proof for the potential of biomining,^54,55^ works because copper-containing sulfidic ore supplies the energy needed for its own breakdown. However, this is particular to sulfidic ores rather than a general result about biomining. Every other target of biomining that we discuss does not carry its own energy, which drives us to heterotrophic organisms like *G. oxydans*, which means buying carbon. This is the single largest uncertainty in our research program, and is particularly acute for accelerated weathering.^125^

While almost any success in biomining is a good thing, success in accelerated weathering faces an uncompromising standard. Natural weathering removes anthropogenic CO_2_ over tens to hundreds of thousands of years,^120^ but the problem needs to be solved in approximately 100 years.^113^ Thus, a process that accelerates weathering even ten-fold is still a failure.

The first answer to this problem is that there are non-acid mechanisms for mineral and metal interactions that could dramatically reduce the amount of carbon needed for biomining and accelerated weathering. However, we have not come close to fully cataloguing the full suite of mechanisms, nor the genes that encode them. Because we have not yet fully explored them, we have no idea of how effective they could be at reducing the amount of carbon needed in biomining and accelerated weathering. The second approach is that we could dramatically improve the carbon supply through electromicrobial production.

### 4.2. Electromicrobial production

EMP is the area of our program where we are most confident about the performance ceiling. However, how we approach this ceiling at large scale remains a huge challenge, which we tackle in a recent preprint.^183^ We conclude that a development program for electromicrobial production must not only be easy to develop in the lab, but it must also face only shallow valleys of death on its way to achieving its ultimate ceiling. No approach to EMP that we are aware of has both of these features. But, we believe that if improvements in CO_2_ to formate reduction continue as they have over the past decade, then EMP systems that use formate for both carbon and electron delivery may be the right route. Future theoretical work must include the energy costs of operating high-temperature hydrogen electrolyzers, the cooling costs of formate electrolyzers, the costs of direct air capture, and a direct comparison with electrochemical CO_2_ reduction followed by Fischer–Tropsch synthesis of complex molecules.

### 4.3. Highly engineerable chassis organisms

Our research program rests on the idea that engineerability unlocks all of our ambitions. The first risk is that the natural competence mechanism that we propose is tightly locked down, and we may not be able to unlock this in a reasonable amount of time.

The second risk is the bottleneck of genetic screening. Our Arsenazo-III bioleaching assay is a good illustration: even though it is far more useful than a simple acid-production, it is time-consuming and expensive.^125^

Third, and most seriously, the organism we want to build may sit in a fitness minimum rather than a maximum. A cell devoting high flux to a single product at high efficiency achieves no reproductive benefit. This inverts the usual logic of directed evolution, and it means that the most powerful tool we have for improving biological function works against us rather than for us.

### 4.4. What would change our minds?

Our research program builds upon itself in series. Knockout Sudoku enabled the discovery of the electron uptake genes, which enabled calculation of a credible efficiency ceiling for EMP, which justified the chassis program, and EMP at scale could relieve the feedstock constraint on biomining and weathering. As almost every grant review panel reminds young scientists, these serial dependencies multiply risk rather than average it. Counter to that, the economics run in a loop rather than a chain, since biomining supplies the critical minerals for the electronics that generate the electricity that powers EMP, so partial success in one area propagates to the others.

Finally, what would make us declare this program a failure, or radically change direction? We cannot honestly name the thresholds in advance, because establishing where the ceilings of biological performance are is by itself a major undertaking. What we can commit to is rigor in making those assessments and a willingness to act on them when they are unwelcome. We have done this before. Our analysis of the carbon demand of bio-accelerated weathering^125^ returned a result we did not want, and it is the reason electromicrobial production became so urgent. Determining whether to proceed is precisely why the basic science in this article was conducted, and a negative answer obtained early is worth considerably more than a positive one obtained late.

## 5. Future directions and conclusion

### 5.1. What's next in biomining?

We believe that the route to developing biomining processes for metals other than copper and gold and accelerated weathering for CO_2_ sequestration passes through much better understanding of the mechanisms used by microorganisms to interact with metals and minerals. But, even if we can understand these mechanisms and the genes that encode them through a Microbe-Mineral Atlas (MMA), how can we harness them? While we do not completely rule out the possibility that microbes can be discovered and cultivated that are well-suited to bioprocesses for the extraction and separation of many of the critical metals that are so essential for modern civilization, we do not believe that this has a high probability, nor do we believe that we should rely upon this. Instead, we believe that the most promising approach is the construction of bespoke organisms tailored for each metal and mineral type that we want to mine that contain as yet unseen in nature combinations of natural genes from the MMA, possibly with genes encoding *de novo* proteins inspired by its findings. But, how do we know which genes to include in these bespoke organisms? We believe our current state of knowledge of the genetics of microorganisms, especially those from environments that are extreme to us, makes answering this question immediately almost impossible. But, we do not believe that this task is impossible, or even just a possibility far in the future. We believe that solving this problem lies in imaginative experimentation to discover new, never-seen-before gene functions. Doing this will be an enormous challenge. Not only will we need imaginative new high-throughput experiments to test for the transformation of metals and minerals, but we will also have to contend with the likelihood that many of the genes that we wish to discover are natively active under unusual conditions of pH, pressure, temperature, salinity and metal concentrations.

### 5.2. What's next in electromicrobial production and highly engineerable microbes?

Unlike in biomining, we do not believe that the route to achieving predicted extremely high conversion efficiencies between electricity, CO_2_ and complex molecules lies in a much better understanding of the fundamental biophysics and genetics of this process. While this may sound arrogant, we believe that we understand the modules of this process (CO_2_ fixation, extracellular electron uptake and product synthesis) at least reasonably well (in contrast to biomining, where we do not even understand the genetics, let alone the mechanism of mineral and metal interactions). Furthermore, the basic process of EMP has been demonstrated, but at nowhere close to theoretical maximum efficiency at large scale.

We think that progress in this area, and achieving high energy conversion efficiency from CO_2_ to a single product like a biofuel, demands much better integration of the modules of EMP. But, we believe that asking these modules to work together to simultaneously deliver high energy conversion efficiency and a high flux of energy and matter into a single product asks the cell to do something deeply unnatural. The goal of a cell is to make the wide range of compounds it needs to duplicate itself, and the emergence of true factory-like behavior has to work against the most powerful tool for improving biological function: evolution by natural or laboratory selection, as factory-like behavior has little obvious benefit to the organism's fitness and reproduction and could be actively harmful for the cell. If we are to build a cell in this state, then achieving it may not be possible with traditional directed evolution that relies upon gains in whole organism fitness. Instead, we believe that constructing this organism will have to rely much more heavily upon *de novo* genome synthesis. Furthermore, unlike in a naturally occurring organism which exists in a (at least local) fitness maximum and where most mutations reduce fitness, this high-flux high-efficiency factory organism might exist in a fitness minimum, and any mutations will increase reproductive fitness. This means that in addition to innovations in the design of a genome, we will need to couple this with much higher genome replication fidelity than is seen in naturally occurring organisms.

## 6. Conclusion

We believe that achieving our goals in sustainability and synthetic biology requires that engineering goes hand-in-hand with basic biological discovery. We do not believe that the dominant paradigm of science of the past 80 years (the Endless Frontier^184^), that basic science must precede engineering, is the right one. We believe that an older paradigm, use-inspired basic research, where engineering and discovery go hand in hand,^37^ much closer to the approach followed by innovators like Louis Pasteur and the Wright brothers,^185^ is the right approach. Achieving these goals could take years, if not decades, but success cannot permanently remain decades in the future. Tackling these problems requires a sustained urgency, and more importantly a sustained search for shortcuts, new knowledge, and new insights that will bring these to reality much sooner.

## Author contributions

G. V.: conceptualization, visualization, writing – original draft, and writing – review and editing. B. B.: conceptualization, funding acquisition, supervision, visualization, writing – original draft, and writing – review and editing.

## Conflicts of interest

Much of the work presented here has been commercialized, or we plan to commercialize it. B. B.'s lab launched REEgen, Forage Evolution, and Gigatonne Bio, and is incubating Biolithica. B. B. co-founded and holds equity in Forage Evolution and Gigatonne Bio, contributes to REEgen, which licenses engineered biomining organisms described in this article, and receives research sponsorship from Biolithica. G. V. declares no conflicts of interest.

## Supplementary Material

CC-OLF-D6CC01792D-s001

CC-OLF-D6CC01792D-s002

## Data Availability

No primary research results, software or code have been included and no new data were generated or analysed as part of this review. The supplementary information (SI) contains information on the rationale behind certain figures, and the background data for the relationship between the quality of life in various countries and energy consumption. See DOI: https://doi.org/10.1039/d6cc01792d.
