# Supplementary material for: Synthetic biology for sustainable abundance: biomining, electromicrobial production, and hyper-engineerable microbes
Source: Chem Commun (Camb). 2026 Oct 22. Online ahead of print. doi: 10.1039/d6cc01792d (PMC13752747; doi:10.1039/d6cc01792d)
Supplement: Supplementary file 1 [file CC-OLF-D6CC01792D-s001.pdf]

Supplementary Information (SI) for Chemical Communications.
This journal is © The Royal Society of Chemistry

Supplementary Information for:

Applying Biology to the Challenge of Sustainable Abundance

Gretchen Vengerova[1] and Buz Barstow[1,†]

[1]Department of Biological and Environmental Engineering, Cornell University, Ithaca, NY 14853, USA.

†Corresponding author:

Buz Barstow, 228 Riley-Robb Hall, Cornell University, Ithaca, NY 14853; bmb35@cornell.edu.

Supplementary Tables

Table S1. Critical metals and example applications in sustainable energy, defense, and computing.

Supplementary Notes

Note S1. Total CO_2 addition to the atmosphere since the start of the Industrial Revolution.

Supplementary Tables

**Metal**	***Z***	**Symbol**	**Criticality**	**Defense Applications**	**Sustainability Applications**	**Primary Mineral Host(s) (primary mined is first)**	**Mineral Type(s)**
Lithium	3	Li	High	Battery systems for vehicles and portable equipment; optoelectronic devices for communications systems.	Li-ion batteries for EVs and grid storage; lithium-aluminosilicate glass-ceramics; Li-based CO2 sorbents (Li_4SiO_4, Li_2ZrO_3) for high-temperature carbon capture.	Spodumene; brine deposits	Silicate (pyroxene); evaporite
Aluminum	13	Al	High	Aircraft and missile construction; ground vehicles; equipment housings.	Lightweighting of vehicles and aircraft (fuel efficiency); solar panel frames and mounting; HVDC transmission conductors; highly recyclable at ~5% of primary energy.	Bauxite; feldspar	Hydroxide/ oxide; silicate (tectosilicate)
Scandium	21	Sc	Critical	Lightweight aluminum-scandium alloys for aircraft and specialized equipment.	Al-Sc alloys for lightweight transport and hydrogen storage tanks; scandia-stabilized zirconia electrolytes for solid oxide fuel cells and electrolyzers.	Byproduct of Ti, Zr, REE ores; thortveitite	Oxide, phosphate, carbonate; silicate
Titanium	22	Ti	High	Aircraft construction; submarine components; armor; corrosion-resistant components.	Corrosion-resistant components for desalination and geothermal plants; electrolyzer and fuel-cell bipolar plates; TiO_2 photocatalysts for water and air remediation.	Ilmenite; rutile	Oxides
Chromium	24	Cr	Moderate	Corrosion-resistant alloys; hardening applications; protective coatings.	Stainless steels for long-life process equipment and water infrastructure; Fe-Cr-Al alloys for high-temperature heat exchangers; corrosion resistance in CCS and electrolyzer systems.	Chromite	Oxide (spinel)
Cobalt	27	Co	High	High-temperature alloys; magnetic applications; batteries.	Li-ion cathodes (NMC, LCO) for EVs and storage; Fischer-Tropsch catalysts for synthetic and sustainable aviation fuels; hydrodesulfurization catalysts.	Cobaltite; carrolite	Sulfides
Nickel	28	Ni	Moderate	Superalloys for jet engines; high-temperature applications; naval systems (INCONEL, NIMONIC, NAB).	High-nickel Li-ion cathodes; Ni-based electrodes and catalysts for alkaline water electrolysis; stainless steels and superalloys for geothermal and nuclear plant components.	Pentlandite; garnierite	Sulfide; silicate
Copper	29	Cu	High	Electrical wiring and circuits; electromagnetic shielding; communication systems. Essential for all electrical systems.	Electrical grid expansion and electrification; EV motors and wiring; wind turbine generators; solar PV interconnects; heat exchangers for heat pumps.	Chalcopyrite; bornite	Sulfides
Gallium	31	Ga	Critical	Gallium arsenide semiconductors for radar; night vision equipment; display technology; high-frequency military communications; satellite systems; electronic warfare systems.	GaN power electronics for efficient inverters and EV chargers; GaAs and CIGS thin-film solar cells; LED lighting.	Sphalerite byproducts from Al and Zn ores	Sulfide
Germanium	32	Ge	Critical	Infrared optics and thermal imaging; night vision systems; fiber optic communications; satellite solar cells; radar; electronic countermeasures.	Ge substrates for high-efficiency multijunction solar cells; fiber optic networks for low-energy data transmission; IR optics for industrial emissions monitoring.	Sphalerite byproducts of Zn ores and coal	Sulfide
Arsenic	33	As	Critical	Infrared systems; high-frequency electronics for radar and communications.	GaAs photovoltaics and power electronics (as compound); semiconductor dopant for efficient electronics; InAs/GaInAsP photodetectors for environmental sensing.	Arsenopyrite; realgar	Sulfide
Yttrium	39	Y	Critical	Radar systems; laser applications; high-temperature superconductors; medical imaging.	Yttria-stabilized zirconia electrolytes for solid oxide fuel cells and electrolyzers; thermal barrier coatings for higher-efficiency gas turbines; LED phosphors.	Xenotime; monazite	Phosphate
Silver	47	Ag	High	High-conductivity electronics; radar systems; communication equipment.	Front-contact metallization in silicon PV cells; high-efficiency electrical contacts and switching; antimicrobial water treatment.	Argentite; acanthite; native silver	Sulfide; sulfide; native metal

Indium	49	In	Critical	Indium tin oxide (ITO) for transparent conductors; cockpit displays and avionics touch screens; helmet-mounted displays; infrared detectors (InSb, InAs); low-temperature solders.	ITO transparent conductors for solar cells and efficient displays; CIGS thin-film PV; InGaN LEDs for low-energy lighting.	Sphalerite byproduct of Zn ores	Sulfide
Antimony	51	Sb	Critical	Bullet hardening (alloyed with lead); tracer rounds and pyrotechnic compositions; explosive primers; flame retardants in electronics and body armor; infrared detectors; thermoelectric cooling; optical equipment.	Lead-acid battery grids for stationary storage; Sb-based liquid-metal batteries for grid storage; flame retardants extending product life; clarifying agent in solar glass.	Stibnite	Sulfide
Lanthanum	57	La	Moderate	Night vision goggles; camera lenses.	NiMH battery anodes for hybrid vehicles; fluid catalytic cracking catalysts; La-based hydrogen storage alloys ($LaNi_5$).	Monazite; bastnäsite	Phosphate; carbonate
Cerium	58	Ce	Moderate	Glass polishing; UV-absorbing applications.	Automotive three-way catalysts and diesel oxidation catalysts; ceria-based oxygen carriers for solar thermochemical fuel and chemical looping; glass polishing with recycling potential.	Monazite; bastnäsite	Phosphate; carbonate
Praseodymium	59	Pr	Moderate	Aircraft engine alloys; specialized glass applications; high-strength permanent magnets.	Nd-Pr permanent magnets for wind turbine generators and EV traction motors; Pr-doped glass and ceramics.	Monazite; bastnäsite	Phosphate; carbonate
Neodymium	60	Nd	Critical	High-strength permanent magnets in missiles aircraft and drones; precision motors.	NdFeB permanent magnets for direct-drive wind turbines, EV traction motors and efficient industrial motors.	Monazite; bastnäsite	Phosphate; carbonate
Samarium	62	Sm	High	High-temperature samarium-cobalt magnets for actuators; radar.	SmCo magnets for high-temperature motors and generators in EVs and aerospace; Sm-doped ceria electrolytes for intermediate-temperature SOFCs.	Monazite; bastnäsite	Phosphate; carbonate
Europium	63	Eu	High	Red phosphors in displays; secure communication systems; nuclear reactor moderators.	Red phosphors for energy-efficient LED and fluorescent lighting; Eu-doped phosphors for solar-spectrum conversion coatings.	Monazite; bastnäsite	Phosphate; carbonate
Gadolinium	64	Gd	High	Neutron detection systems.	Magnetocaloric refrigeration (non-vapor-compression cooling); Gd-doped ceria electrolytes for SOFCs; MRI contrast agents.	Monazite; bastnäsite	Phosphate; carbonate
Terbium	65	Tb	Critical	Green phosphors for night vision; magnetostrictive sonar systems; guidance systems; radar.	Dy/Tb-substituted NdFeB magnets for high-temperature EV motors and wind generators; green phosphors for efficient lighting; Terfenol-D actuators.	Monazite; bastnäsite	Phosphate; carbonate
Dysprosium	66	Dy	Critical	High-temperature permanent magnets.	Coercivity additive in NdFeB magnets for EV traction motors and wind turbine generators operating at elevated temperature.	Monazite; bastnäsite	Phosphate; carbonate
Holmium	67	Ho	Low	Precision laser systems; nuclear reactor moderator.	Dopant for high-power fiber and solid-state lasers used in medical and industrial processing; magnetic flux concentrators.	Monazite; bastnäsite	Phosphate; carbonate
Erbium	68	Er	Moderate	Fiber optic communications; range-finding lasers.	Erbium-doped fiber amplifiers enabling low-energy long-haul telecommunications; upconversion phosphors for enhanced PV spectral response.	Monazite; bastnäsite	Phosphate; carbonate
Thulium	69	Tm	Moderate	Portable X-ray sources; medical lasers for field operations.	Thulium fiber lasers for efficient industrial materials processing; dopant in high-temperature superconductors; portable X-ray sources for medical access.	Monazite; bastnäsite	Phosphate; carbonate
Ytterbium	70	Yb	High	Fiber optic amplifiers.	Yb-doped fiber lasers (high wall-plug efficiency) for cutting, welding and additive manufacturing; Yb dopant in high-efficiency solar-cell upconversion layers.	Monazite; bastnäsite	Phosphate; carbonate
Lutetium	71	Lu	Low	Optical lenses and X-ray phosphors for specialized medical imaging.	Lu-based scintillators (LSO/LYSO) for PET medical imaging; catalysts in petroleum cracking; Lu-doped phosphors.	Monazite; bastnäsite	Phosphate; carbonate

Tantalum	73	Ta	Critical	High-temperature electronics for missiles; high-voltage capacitors for mission-critical electronics including drones; corrosion-resistant applications; electronic warfare systems.	Miniaturized high-reliability capacitors extending electronics lifetime; corrosion-resistant heat exchangers for chemical and acid processing; biocompatible medical implants.	Tantalite; columbite, rutile	Oxides
Tungsten	74	W	Critical	Armor-piercing projectiles; tank armor and protective systems; kinetic energy penetrators; X-ray shielding; high-temperature components.	Cemented carbide tooling for efficient machining and mining; high-temperature electrodes for arc furnaces; tungsten oxide electrochromic windows for building energy savings.	Wolframite; scheelite	Oxides
Platinum	78	Pt	Moderate	Specialized electronic components; sensors; and catalysts.	PEM fuel-cell and electrolyzer catalysts for green hydrogen; automotive emission-control catalysts; nitric acid and silicone process catalysts.	Sperrylite; native platinum	Sulfide, Arsenide; native metal
Gold	79	Au	High	High conductivity for critical electronic components and connectors and circuits; corrosion-resistant applications.	Reliable low-resistance contacts in electronics (extended service life); Au nanoparticle catalysts for low-temperature CO oxidation; highly recycled from e-waste.	Gold-bearing pyrite; native gold; calaverite	Sulfide; native metals; telluride
Bismuth	83	Bi	Critical	Non-toxic replacement for lead in ammunition; low-melting alloys for safety systems; specialized solders.	Non-toxic replacement for lead in solders, ammunition and plumbing brass; Bi-based catalysts for electrochemical CO_2 reduction to formate; thermoelectric (Bi_2Te_3) waste-heat recovery.	Bismuthinite; native bismuth	Sulfide; native metal

Table S1. Critical metals and example applications in sustainable energy, defense, and computing.

Supplementary Notes

Note S1. Ratio Between Power Use of Civilization and Earth's Biosphere

The most recent estimate of power use by human civilization is 592.22 exajoules (EJ) in 2024 [EnergyInst2025a], corresponding to an instantaneous average power consumption of 18.80 terawatts (TW). This corresponds to an increase of almost 12% from 529.47 EJ per year or 16.79 TW since the last time we examined this issue in 2016 [Adesina2017a]. This corresponds to an average annual growth rate of 1.4%. In contrast, the total power use of the biosphere is ≈ 130 TW [Steger2005a]. Thus, the biosphere channels ≈ 7× as much power as is used by human civilization.

Supplementary References

[Adesina2017a] O. Adesina, I.A. Anzai, J.L. Avalos, and B. Barstow, "Embracing Biological Solutions to the Sustainable Energy Challenge", *Chem* 2, 20–51 (2017). doi:10.1016/j.chempr.2016.12.009.

[EnergyInst2025a] *2025 Statistical Review of World Energy*, Energy Institute, London (2025). https://www.energyinst.org/statistical-review.

[Steger2005a] U. Steger *et al.*, *Sustainable Development and Innovation in the Energy Sector,* Springer (2005).
